# Upregulation of LAG3 modulates the immune imbalance of CD4^+^ T-cell subsets and exacerbates disease progression in patients with alveolar echinococcosis and a mouse model

**DOI:** 10.1371/journal.ppat.1011396

**Published:** 2023-05-12

**Authors:** Dewei Li, Abidan Ainiwaer, Xuran Zheng, Maolin Wang, Yang Shi, Zibigu Rousu, Xinling Hou, Xuejiao Kang, Muesier Maimaiti, Hui Wang, Jing Li, Chuanshan Zhang

**Affiliations:** 1 State Key Laboratory of Pathogenesis, Prevention and Treatment of High Incidence Diseases in Central Asia, Clinical Medicine Institute, The First Affiliated Hospital of Xinjiang Medical University, Urumqi, Xinjiang, China; 2 Basic Medical College, Xinjiang Medical University, Urumqi, Xinjiang, China; 3 Xinjiang Key Laboratory of Echinococcosis, Clinical Medicine Institute, The First Affiliated Hospital of Xinjiang Medical University, and WHO Collaborating Centre on Prevention and Case Management of Echinococcosis, Urumqi, Xinjiang, China; Uniformed Services University: Uniformed Services University of the Health Sciences, UNITED STATES

## Abstract

Infection with the cestode *Echinococcus multilocularis* (*E*. *multilocularis*) causes alveolar echinococcosis (AE), a tumor-like disease predominantly affecting the liver but able to spread to any organ. T cells develop functional defects during chronic *E*. *multilocularis* infection, mostly due to upregulation of inhibitory receptors such as T-cell immunoreceptor with immunoglobulin and immunoreceptor tyrosine-based inhibitory motif domains (TIGIT) and programmed death-1 (PD-1). However, the role of lymphocyte activation gene-3 (LAG3), an inhibitory receptor, in AE infection remains to be determined. Here, we discovered that high expression of LAG3 was mainly found in CD4^+^ T cells and induced regulatory T cells (iTregs) in close liver tissue (CLT) from AE patients. In a mouse model of *E*. *multilocularis* infection, LAG3 expression was predominantly found in T helper 2 (Th2) and Treg subsets, which secreted significantly more IL-4 and IL-10, resulting in host immune tolerance and disease progression at a late stage. Furthermore, LAG3 deficiency was found to drive the development of effector memory CD4^+^ T cells and enhance the type 1 CD4^+^ T-cell immune response, thus inhibiting metacestode growth *in vivo*. In addition, CD4^+^ T cells from LAG3-deficient mice produced more IFN-γ and less IL-4 when stimulated by *E*. *multilocularis* protoscoleces (EmP) antigen *in vitro*. Finally, adoptive transfer experiments showed that LAG3-knockout (KO) CD4^+^ T cells were more likely to develop into Th1 cells and less likely to develop into Tregs in recipient mice. Our work reveals that high expression of LAG3 accelerates AE disease progression by modulating the immune imbalance of CD4^+^ T-cell subsets. These findings may provide a novel immunotherapeutic strategy against *E*. *multilocularis* infection.

## Author summary

Alveolar echinococcosis (AE) is a parasitic disease with high mortality caused by *E*. *multilocularis* infection. Our previous studies confirmed that T-cell-mediated cellular immunity, especially that mediated by CD4^+^ T cells, plays a key role in limiting metacestode growth. Here, we report that high LAG3 expression in liver-infiltrating CD4^+^ T cells is associated with their immune imbalance in AE patients and a mouse model of later-stage disease. High expression of LAG3 promotes the formation of Th2 cells and Tregs, and LAG3 deficiency induces a shift in the immune response to the Th1 type to delay the growth of metacestodes in chronic *E*. *multilocularis* infection. These findings suggest that LAG3 blockade may be an effective immune strategy for the treatment of AE patients.

## Introduction

Alveolar echinococcosis (AE) is a serious zoonotic parasitic disease caused by the cestode (metacestode) *Echinococcus multilocularis* (*E*. *multilocularis*) that mimics slow-growing liver cancer. This disease is usually fatal in some regions or countries with poor sanitation conditions because the mortality rate of patients without adequate treatment is more than 90% within 10–15 years [1]. The main factor affecting *E*. *multilocularis* parasitism is the immune status of the host, and host immunosuppression affects the development of *E*. *multilocularis* infection in patients with AE and in an experimental model. Therefore, understanding the pathogenic mechanisms of *E*. *multilocularis* infection may provide a basis for its elimination or the invention of intervention measures.

T lymphocytes are essential for immune responses against *E*. *multilocularis* [2]. An increased risk of *E*. *multilocularis* infection was observed in immunologically hyporesponsive populations, especially in HIV-positive individuals, which indicates that CD4^+^ T cells seem to be crucial [3,4]. However, obtaining an understanding of how CD4^+^ T lymphocytes regulate the disease process is proving to be a daunting challenge. In the case of AE, hepatic histopathological and granulomatous responses are critically regulated by T helper 1 (Th1)/Th2 responses [2,5,6]. During the acute phase of *E*. *multilocularis* infection, the liver has a high Th1 response to parasite antigens, but subsequently, the antigens induce a sustained Th2 response that mediates granuloma formation and fibrosis [2,7]. Meanwhile, regulatory T cells (Tregs) are recruited to the liver and exert a stronger immunosuppressive effect to limit the formation of inflammatory granulomas and fibrosis [5,8]. This persistent effect induces an immune imbalance of CD4^+^ T cells in the liver. Moreover, previous studies have demonstrated that immune inhibitory receptors [e.g., programmed cell death protein-1 (PD-1), T-cell immunoreceptor with immunoglobulin (Ig) and immunoreceptor tyrosine-based inhibitory motif (ITIM) domains (TIGIT)] participate in the dysfunction and imbalance of T-cell subsets during chronic *E*. *multilocularis* or hepatitis B virus (HBV) infection to maintain immune tolerance, thus accelerating disease progression [6,9–12]. Consequently, the role of coinhibitory receptors in AE has attracted much attention.

As a member of the immune inhibitory receptor family, lymphocyte activation gene-3 (LAG3 or CD223) is mainly expressed in activated T cells and inhibits T-cell proliferation and effector function by recognizing conformationally stable peptides and MHC class II (pMHCII) complexes rather than fibrinogen-like protein 1 (FGL-1) [13–15]. LAG3 can also move to the immunological synapse and bind to the T-cell receptor (TCR)-CD3 complex, reduce the potential of hydrogen (pH) of the immune synapse and drive the dissociation of the tyrosine kinase lymphocyte cell kinase (Lck) from its coreceptor, thereby limiting T-cell activation [16]. Functionally, LAG3 signaling restricts the proliferation and effector of conventional T (T_conv_) cells and promotes the differentiation and inhibitory function of CD4^+^CD25^+^Foxp3^+^ Tregs [17]. When LAG3 is not present, there is decreased proliferation and inhibition of Foxp3^+^ Tregs, resulting in increased CD4^+^ T-cell expansion and differentiation into Th1 cells [17,18]. However, one study found the opposite result, suggesting that LAG3 may still intrinsically limit Treg proliferation and function [19]. This contradictory result complicates the assessment of the role of LAG3 in Tregs. Indeed, in our previous studies, we demonstrated high LAG3 expression levels in exhausted CD4^+^ T cells during chronic *E*. *multilocularis* infection [2]. Accordingly, it is critical to define whether LAG3 expression in T_conv_ cells or Tregs affects the progression of AE. However, definitive evidence that LAG3 mediates chronic *E*. *multilocularis* parasitism by affecting CD4^+^ T-cell subsets is limited.

In this study, we analyzed the LAG3 expression profile in intrahepatic and peripheral T cells in AE patients. We confirmed that LAG3 is mainly distributed on CD4^+^ T cells and Tregs. In a mouse model of *E*. *multilocularis* infection, LAG3 expression was predominantly found in Th2 and Treg subsets that induce host immune tolerance, which significantly promoted disease progression at a late stage. In addition, LAG3 deficiency promoted the Th1-type immune response of CD4^+^ T cells *in vivo* and *in vitro* and thus inhibited metacestode growth. Nonetheless, LAG3 deficiency did not significantly delay disease progression at a middle stage of *E*. *multilocularis* infection. Subsequently, we found that CD4^+^ T cells from LAG3-knockout (KO) donors secreted more IFN-γ and less IL-4 in *E*. *multilocularis* infection by two modeled adoptive transfer protocols. Moreover, LAG3 deficiency inhibited the differentiation of naive CD4^+^ T cells into Tregs while promoting Th1 differentiation. Our study demonstrates that LAG3 accelerates AE disease progression by modulating the immune imbalance of CD4^+^ T-cell subsets, and the effect of LAG3 on disease remission is predominantly mediated by Th1 cells.

## Results

### High LAG3 expression in liver-infiltrating CD4^+^ T cells and Tregs of AE patients

**Fig 1:**
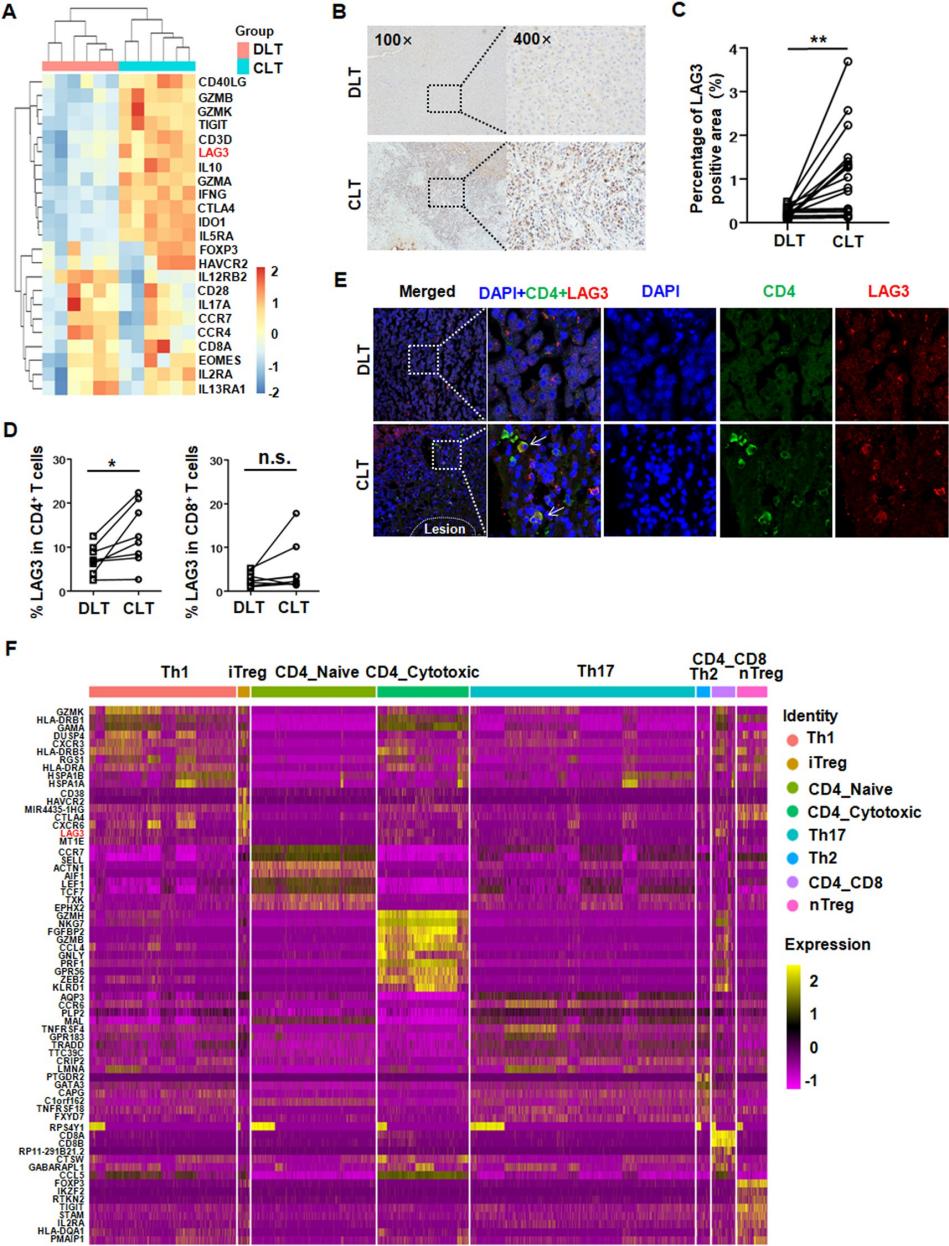
LAG3 expression is up-regulated in infiltrating CD4^+^ T cells and Treg cells from liver tissue of AE patients. **(A)** Heat map presentation of differentially expressed genes related to T cell function, and selected up-regulated genes including negative and positive regulation of T cell activation. **(B)** Representative immunohistochemical staining of LAG3 (left panel 100×, enlarged 400× on the right panel) in the liver tissue sections from patients with AE. **(C)** The percentage of positive staining area was calculated to evaluate the expression of LAG3 (n = 19). **(D)** The percentage of liver CD4^+^ or CD8^+^ T cells expressing LAG3 in paired CLT *versus* DLT from AE patients by flow cytometry (n = 8). **(E)** Representative images from immunofluorescence co-staining of DAPI (blue), CD4 (green), LAG3 (red) and merged image on the liver tissue sections from patients with AE (n = 8). Boxed areas show × 200 magnification of histological images. Arrows indicate CD4^+^LAG3^+^ T cells. Lesion is delimited with a white line. **(F)** Heatmap representing relative expression of selected marker genes in each cluster in CLT, DLT and PB from AE patients (n = 4, accession no. HRA000553). CLT, “close” liver tissue; DLT, “distant” liver tissue; PB, peripheral blood. Data were analyzed using paired Student’s t tests or one-way ANOVA test. All data are presented as mean ± SD. *P < 0.05, **P <0.01, n.s., *P* > 0.05.

We first analyzed mRNA arrays of paired liver tissues [close liver tissue (CLT) versus distant liver tissue (DLT)] from six patients with chronic AE (accession no. GSE124362[6]). The array data showed that the elevated expression of genes was associated with T-cell function. Interestingly, several of the enriched genes encoded inhibitory receptors (*LAG3*, *CTLA4* and *TIGIT*), cell factors (*IL-10*, and *IFN-γ*) and transcription factors (*Foxp3*), and the results were displayed in a heatmap (Fig 1A). Immunohistochemical staining was used on a higher number of samples to investigate LAG3 protein expression in liver tissues from AE patients. There were higher numbers of LAG3^+^ cells in the periparasitic area of CLT specimens than in that of DLT specimens (Fig 1B and 1C). Next, we used flow cytometric analysis to examine LAG3 in T cells. The percentage of LAG3^+^CD4^+^ T cells, but not of LAG3^+^CD8^+^ T cells, was significantly higher in CLT than DLT in paired analysis (Fig 1D). LAG3 expression colocalized with CD4^+^ T cells in CLT according to multicolor fluorescent staining of snap-frozen tissue sections (Fig 1E).

To further characterize CD4^+^ T cells, we analyzed a 10× 5’ single-cell sequencing dataset of CD45^+^ immune cells from paired liver tissue and peripheral blood (PB) mononuclear cells from 4 patients with AE (accession no. HRA000553; https://figshare.com/s/f2a4578be27c2cb767f9 [20]). We identified an induced Treg (iTreg) cluster expressing high levels of *CD38*, *HAVCR2*, *MIR4435-1HG*, *CTLA4*, *CXCR6*, *LAG3* and *MT1E* in CLT, DLT and PB (Fig 1F). Uniform manifold approximation and projection (UMAP) plot and violin plot analyses also revealed that LAG3 was mainly expressed by iTregs and natural killer T (NKT) cells (CD4^+^CD8^+^ T cells) (S1 Fig). Taken together with the results of the scRNA-seq analysis, these findings indicate that LAG3 expression was upregulated in infiltrating CD4^+^ T cells and iTregs from the liver tissue of AE patients.

### Upregulation of LAG3 expression in both Th2 and Tregs in a mouse model of *E*. *multilocularis* infection at a late stage

**Fig 2:**
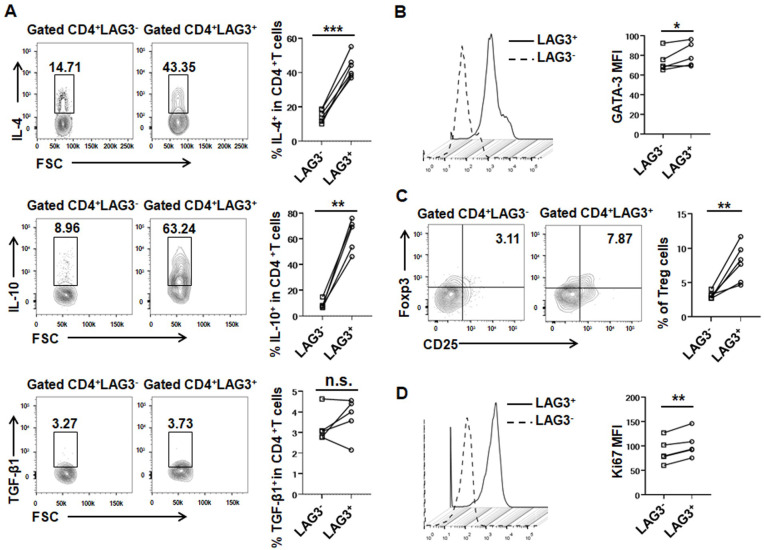
LAG3 is mainly expressed on Th2 and Treg cells in liver of *E*. *multilocularis*-infected mice after weeks 24 of infection. **(A)** Representative flow cytometry plot and percentage of IL-4, IL-10 and TGF-β1 production by CD4^+^ T cells in liver from mice after 24 weeks of infection (5–6 mice per group). **(B)** MFI of GATA3 expression by LAG3^+^ or LAG3^-^ CD4^+^ T cells in liver from mice after 24 weeks of infection (5 mice per group). **(C)** Representative flow cytometry plot and percentage of Treg cells (CD4^+^CD25^+^Foxp3^+^) by LAG3^+^ or LAG3^-^ CD4^+^ T cells in the liver from mice after 24 weeks of infection (6 mice per group). **(D)** MFI of Ki67 expression by LAG3^+^ or LAG3^-^ CD4^+^ T cells in liver from mice after 24 weeks of infection (5 mice per group). All data are presented as mean ± SD. *P < 0.05, **P < 0.01, ***P < 0.001, n.s., *P* > 0.05.

We previously reported that LAG3 expression was mainly distributed in hepatic and splenic CD4^+^ T cells in a mouse model of *E*. *multilocularis* infection [21]. Subsequently, we investigated LAG3 expression on CD4^+^ T-cell subsets in the liver and spleen of mice after 24 weeks of infection. LAG3^+^CD4^+^ T cells expressed significantly more IL-4 and IL-10 than their LAG3^-^ counterparts, whereas there was no significant change in TGF-β1 production in the liver and spleen (Figs 2A and S2A). Moreover, LAG3^+^CD4^+^ T cells expressed significantly higher GATA-3 levels than LAG3^-^CD4^+^ T cells, showing enhanced Th2 response (Figs 2B and S2B). In addition, the percentage of liver- and spleen-infiltrating Tregs in LAG3^+^CD4^+^ T cells was significantly increased (Figs 2C and S2C). We also analyzed the proliferation of CD4^+^ T cells based on the expression of Ki67 and found that LAG3^+^CD4^+^ T cells proliferated more than LAG3^-^CD4^+^ T cells after 24 weeks of infection (Figs 2D and S2D). Collectively, the above results indicated that LAG3 expression was enriched in Th2 and Treg subsets from liver- and spleen-infiltrating CD4^+^ T cells in mice with *E*. *multilocularis* infection.

### LAG3 deficiency delays metacestode growth by enhancing the Th1 immune response in a mouse model of *E*. *multilocularis* infection at a late stage

**Fig 3:**
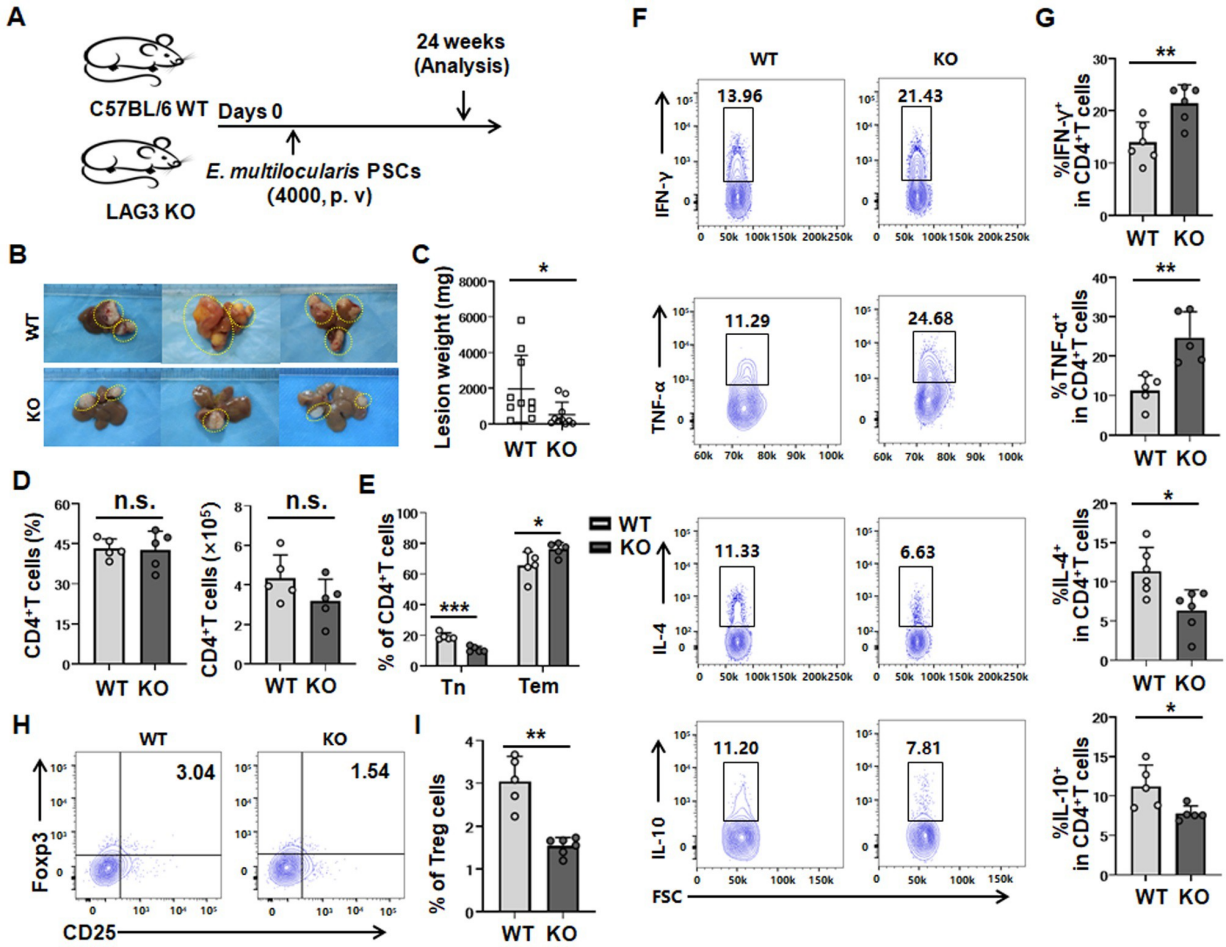
LAG3 deficiency delays disease progression by promoting Th1 cell differention in liver of *E*. *multilocularis*-infected mice after weeks 24 of infection. **(A)** Establishment of a protocol in the late stage of *E*. *multilocularis*-infected mice. **(B)** Representative images of metacestode tissue in the liver from WT and LAG3-KO mice after 24 weeks of infection. Metacestode tissues are circled by the yellow line. **(C)** Lesion weight in the liver from WT and LAG3-KO mice after 24 weeks of infection (10 mice per group). **(D)** Percentage and absolute numbers of CD4^+^T cells in the liver from WT and LAG3-KO mice after 24 weeks of infection (5 mice per group). **(E)** Percentage of Tn and Tem in CD4^+^ T cells in the liver from WT and LAG3-KO mice after 24 weeks of infection (5 mice per group). **(F, G)** Representative flow cytometry plot and percentage of IFN-γ, TNF-α, IL-4 and IL-10 production by CD4^+^ T cells in the liver from WT and LAG3-KO mice after 24 weeks of infection (5–6 mice per group). **(H, I)** Representative flow cytometry plot and percentage of Treg (CD4^+^CD25^+^Foxp3^+^) cells in the liver from WT and LAG3-KO mice after 24 weeks of infection (5–6 mice per group). KO, knockout; WT, wild type; Tn, naive T cells (CD44^-^CD62L^+^); Tem, effector T cells (CD44^+^CD62L^-^). All data are presented as mean ± SD. *P < 0.05, **P < 0.01, ***P < 0.001, n.s., *P* > 0.05.

To further prove the role of LAG3 in CD4^+^ T cells during *E*. *multilocularis* infection, we investigated wild-type (WT) and LAG3-KO mice infected for 24 weeks. We found that LAG3 deficiency inhibited liver metacestode growth. Lesion weight was significantly decreased in LAG3-deficient mice compared to WT mice (Fig 3A–3C). There were no significant differences in the percentages or absolute numbers of liver- and spleen-infiltrating CD4^+^ T cells (Fig 3D). However, there was a significant increase in the percentages of liver- and spleen-infiltrating effector memory CD4^+^ T (Tem) cells (Figs 3E and S3B). Furthermore, significantly increased levels of IFN-γ and TNF-α were produced by liver- and spleen-infiltrating CD4^+^ T cells from LAG3-deficient mice compared to those form WT mice; in addition, lower levels of IL-4 and IL-10 were observed in liver CD4^+^ T cells from LAG3-deficient mice (Figs 3F, 3G, S3C and S3D). Treg infiltration in the liver and spleen of LAG3-deficient mice was also decreased compared to that of WT mice (Figs 3H, 3I, S3E and S3F). The liver Ki67 MFI of LAG3^+^ Tregs was greater than that of their LAG3^-^ counterparts and was lower in LAG3-KO mice than in WT mice after 24 weeks (S4A and S4C Fig). However, there was no significant difference in the spleen (S4B and S4D Fig). Collectively, these results indicated that LAG3 deficiency can enhance antiparasitic immunity by limiting Th2 and Treg responses while promoting the Th1 response at a late stage after 24 weeks of infection.

Moreover, we also assessed WT or LAG3-KO mice at 12 weeks of infection. We found that hepatic metacestode growth was mildly inhibited in LAG3-KO mice, but lesion weight was not significantly different from that in WT mice (S5A and S5B Fig). LAG3 deficiency did not affect the infiltration of CD4^+^ T cells and Tregs or the phenotype of CD4^+^ Tem cells in the liver and spleen (S5C, S5D, S5G, S5H, S6A, S6B, S6E and S6F Figs). However, there were significant increases in the percentages of CD4^+^ T cells and CD4^+^ Tem cells in spleens from LAG3-KO mice after 12 weeks of infection (S6C and S6D Fig). Furthermore, the numbers of CD4^+^ T cells expressing IFN-γ and TNF-α in the liver and spleen were significantly higher in LAG3-deficient mice than in WT mice (S5E, S5F, S6C and S6D Figs). In contrast, significantly decreased levels of IL-4 were produced by liver and splenic CD4^+^ T cells from LAG3-deficient mice (S5E, S5F, S6C and S6D Figs). Interestingly, significantly increased levels of IL-10 were produced by liver and splenic CD4^+^ T cells from LAG3-deficient mice after 12 weeks of infection (S5E, S5F, S6C and S6D Figs). These data showed that unlimited metacestode growth may be related to the high level of IL-10 secretion by CD4^+^ T cells in LAG3-KO mice after a moderate length of infection (12 weeks).

**Fig 4:**
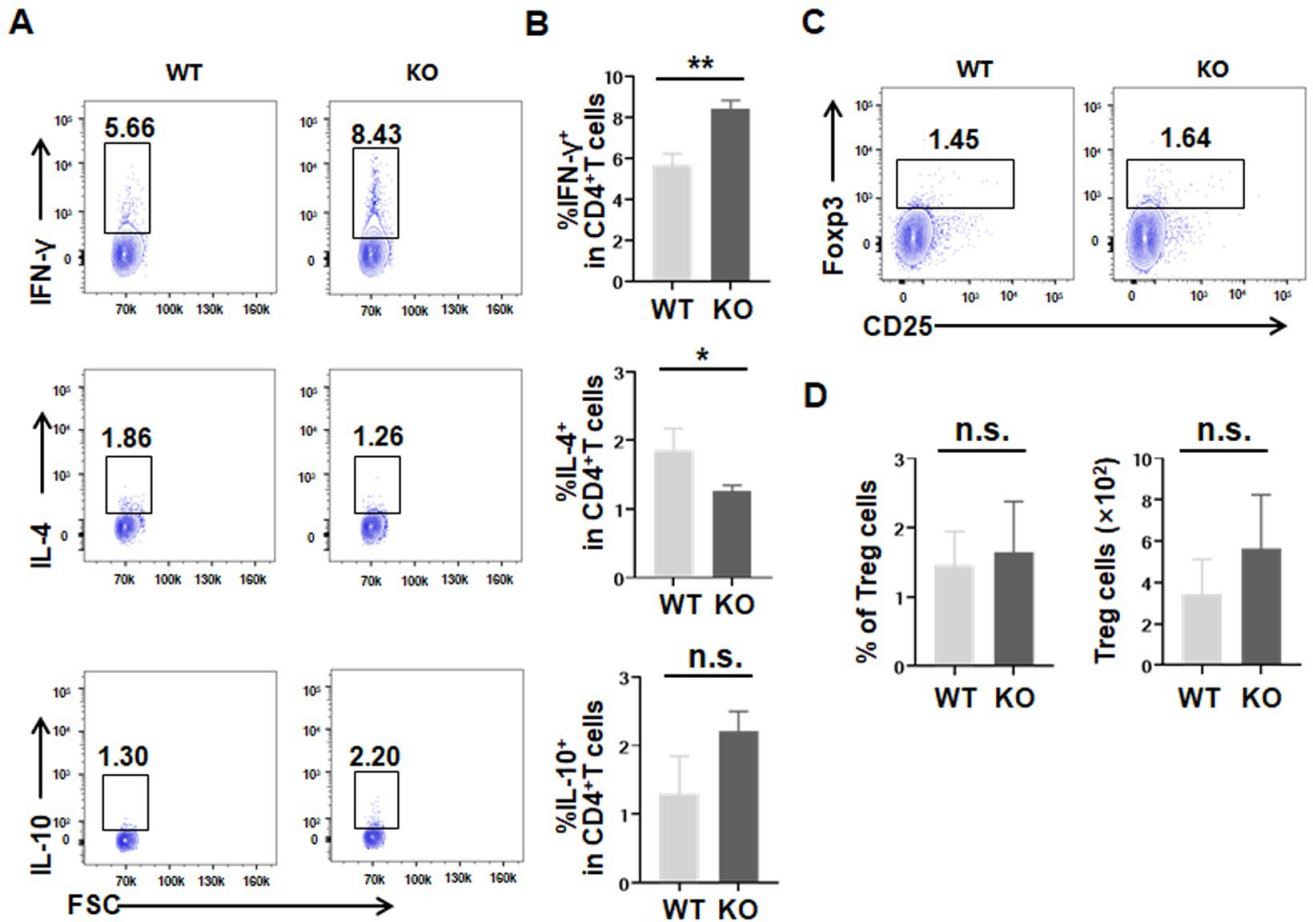
Increased Th1 cytokine production by LAG3-deficient CD4^+^ T cell following *E*. *multilocularis* protoscoleces antigen stimulation *in vitro*. **(A, B)** Representative flow cytometry plot and percentage of IFN-γ, IL-4 and IL-10 production by liver CD4^+^ T cells from WT and LAG3-KO mice with *E*. *multilocularis* protoscoleces antigen stimulation *in vitro*. **(C, D)** Representative flow cytometry plot, percentage and absolute numbers of liver Treg (CD4^+^CD25^+^Foxp3^+^) cells from WT and LAG3-KO mice with *E*. *multilocularis* protoscoleces antigen stimulation *in vitro*. KO, knockout. All data are presented as mean ± SD. *P < 0.05, **P < 0.01, n.s., *P* > 0.05.

To determine whether LAG3 deficiency affects the polarization of CD4^+^ T helper cells, we stimulated liver-isolated lymphocytes with *E*. *multilocularis* protoscoleces (EmP) antigen *in vitro*. The level of IFN-γ in CD4^+^ T cells of LAG3-deficient mice was significantly upregulated compared with that of WT mice, whereas the level of IL-4 was significantly decreased in CD4^+^ T cells of LAG3-deficient mice (Fig 4A and 4B). In addition, there was no significant difference in the level of IL-10 or the percentage of Tregs (Fig 4A–4D). These results highlighted that EmP enhanced Th1-related IFN-γ production in liver-derived LAG3-deficient lymphocytes, mimicking *E*. *multilocularis* infection.

### LAG3-deficient CD4^+^ T cells develop into Th1 cells upon adoptive transfer into *E*. *multilocularis-*infected mice

**Fig 5:**
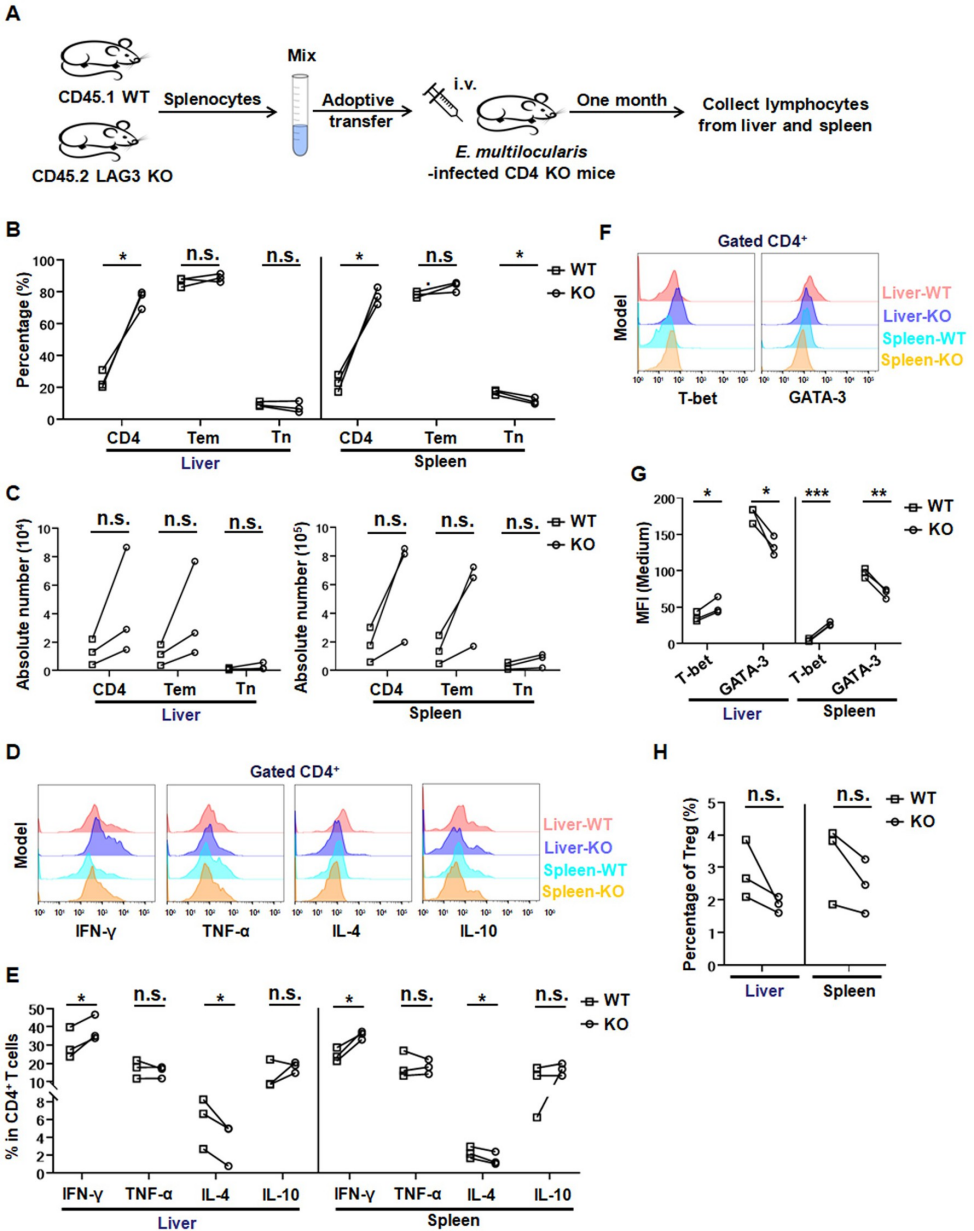
LAG3-deficient CD4^+^ T cell develop into Th1 cells upon adoptive transfer into *E*. *multilocularis* infected CD4 KO mice. **(A)** Establishment of adoptive transfer mouse model. Splenocytes were isolated from WT (CD45.1) and LAG3-KO (CD45.2) mice, mixed, and then transferred to *E*. *multilocularis*-infected CD4-KO recipient mice. **(B, C)** Percentage and absolute number of transferred CD4^+^ T cells, CD4^+^ Tn and CD4^+^ Tem in liver and spleen of *E*. *multilocularis*-infected CD4-KO mice that received adoptive transfer. **(D, E)** Representative flow cytometry plot and percentage of IFN-γ, TNF-α, IL-4, IL-10 production by transferred CD4^+^ T cells in liver and spleen from *E*. *multilocularis*-infected CD4-KO mice. **(F, G)** Representative flow cytometry plot and MFI of T-bet and GATA-3 expression by transfered CD4^+^ T cells in the liver and spleen from *E*. *multilocularis*-infected CD4-KO mice. **(H)** Percentage of Treg cells (CD4^+^CD25^+^Foxp3^+^) in the liver and spleen from *E*. *multilocularis*-infected CD4-KO mice that received adoptive transfer. KO, knockout; WT, wild type. All data are presented as mean ± SD. (n = 3) *P < 0.05, **P < 0.01, ***P < 0.001, n.s., *P* > 0.05.

**Fig 6:**
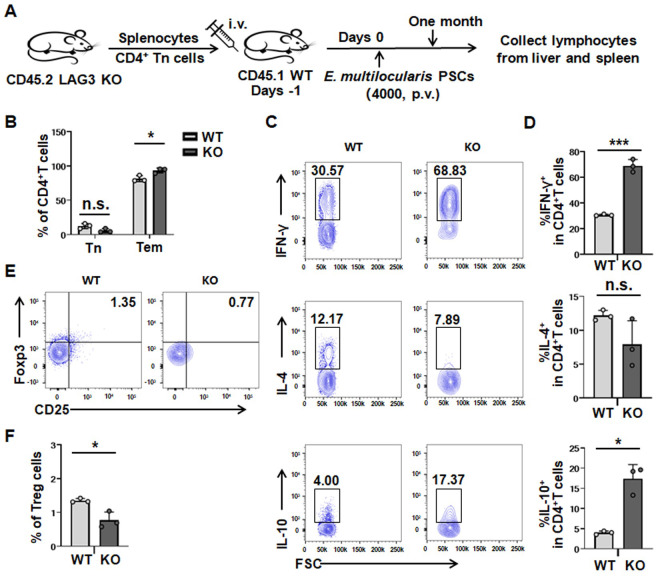
LAG3 deficiency enhance Th1 cells and decrease Treg upon adoptive transfer into wild-type mice followed by *E*. *multilocularis* infection. **(A)** Establishment of adoptive transfer mouse model. CD4^+^ Tn cells were isolated from spleen of LAG3-KO (CD45.2) mice and transferred to WT (CD45.1) recipient mice one day before *E*. *multilocularis* infection. **(B)** Percentage of CD4^+^ Tn and CD4^+^ Tem in the liver from *E*. *multilocularis*-infected WT recipient mice (CD45.1) with transferred by LAG3-KO (CD45.2) cells. **(C, D)** Representative flow cytometry plot and percentage of IFN-γ, IL-4 and IL-10 production by CD4^+^ T cells in the liver from *E*. *multilocularis*-infected WT recipient mice (CD45.1) with transferred by LAG3-KO (CD45.2) cells. **(E, F)** Representative flow cytometry plot and percentage of Treg cells (CD4^+^CD25^+^Foxp3^+^) in the liver from *E*. *multilocularis*-infected WT recipient mice (CD45.1) with transferred by LAG3-KO (CD45.2) cells. KO, knockout; WT, wild type; Tn, naive T cells. All data are presented as mean ± SD. *P < 0.05, ***P < 0.001, n.s., *P* > 0.05.

To better define whether there is a requirement for LAG3 either during the maintenance phase or at the time of priming for *E*. *multilocularis* infection-induced CD4^+^ T helper cell polarization, we modified our adoptive transfer protocol to establish infection in either CD4^+^ T-cell LAG3-KO mice or WT CD45.1 recipients that had been infected 30 days or one day before *E*. *multilocularis* infection (Figs 5A and 6A). Both the proportions and absolute numbers of CD4^+^ T cells and CD4^+^ Tem cells in the liver and spleen were higher in LAG3-KO donor cells than WT donor cells, although the absolute numbers were not significantly different (Fig 5B and 5C). The level of IFN-γ in CD4^+^ T cells from LAG3-KO donors was significantly upregulated compared with that of WT donors, whereas the level of IL-4 was significantly decreased in CD4^+^ T cells of LAG3-KO donors (Fig 5D and 5E). However, CD4^+^ T cells of LAG3-KO donors had similar expression of TNF-α and IL-10 as WT donors (Fig 5D and 5E). In addition, CD4^+^ T cells in the liver and spleen of LAG3-KO donors expressed significantly higher levels of T-bet but lower levels of GATA-3 than those of WT donors (Fig 5F and 5G). The proportion of Tregs in liver and spleen cells from LAG3-KO donor cells was lower than that from WT donor cells, although the difference was not statistically significant (Fig 5H).

Furthermore, we evaluated the role of LAG3 in modulating naive CD4^+^ T-cell polarization *in vivo* by adoptively transferring cells 1 day prior to *E*. *multilocularis* infection. We observed that the proportion of CD4^+^ Tem cells in the liver and spleen of LAG3-KO donor cells was significantly increased compared with of WT donor cells (Figs 6B and S7A). The percentage of IFN-γ- and IL-10-producing CD4^+^ T cells was greatly increased in the liver and spleen of LAG3-KO donor cells, whereas IL-4 expression was decreased in the liver, although the difference was not statistically significant (Figs 6C, 6D, S7B and S7C). We found that the percentage of Tregs in the liver and spleen was significantly reduced in LAG3-KO mice compared with WT mice (Figs 6E, 6F, S7D and S7E). Collectively, the above results demonstrated that LAG3 deficiency inhibited Treg differentiation but promoted Th1 differentiation and IFN-γ expression in response to *E*. *multilocularis* infection.

## Discussion

In general, the liver is the preferred site for many tumors to metastasize and viruses and parasites to colonize, indicating that it has a unique immune environment [22–25]. Inhibitory receptors play a pivotal role in modulating the immune response in liver disease and are mediators of T-cell dysfunction and/or exhaustion in chronic viral and parasitic infections [26,27]. *E*. *multilocularis* infections in humans are located almost exclusively (98%) in the liver [28]. Our recent research showed that T cell factors, especially CD4^+^ T-cell dysfunction or deficiency, accelerated lesion growth in the liver in chronic *E*. *multilocularis* or *Echinococcus granulosus* infection, demonstrating their importance in defense against tapeworm invasion [6]. However, how to modulate T-cell responsiveness as a treatment for AE remains a challenge. Here, we demonstrate that LAG3 affects CD4^+^ T cells to participate in the immune tolerance induced by *E*. *multilocularis* infection and plays a critical role in long-term infection; LAG3 deficiency affects CD4^+^ T-cell skewing, promoting a Th1 phenotype and inhibiting Th2 and Treg induction, which strongly suggests that blocking LAG3 could be a strategy used in future clinical treatment of patients with AE.

Accumulating evidence indicates that high expression of inhibitory receptors (PD-1, CTLA-4, and LAG3) on CD4^+^ T cells is related to decreased secretion of cytokines and proliferation capacity of T cells, such as IFN-γ^+^ Th1, IL-4^+^ Th2, and IL-17A^+^ Th17 cells and IL-10^+^ Tregs [2,29,30]. LAG3, as a CD4-related activation-inducing cell surface molecule, can negatively regulate mitochondrial biogenesis and metabolism in naive CD4^+^ T cells, thereby controlling their phenotypic changes [31]. In addition, LAG3 has previously been described on different CD4^+^ T-cell subsets, including Th1 cells, Tregs, and Tr1 cells [2,17,32,33]. In previous studies, we have shown that at different stages of infection after *E*. *multilocularis* challenge, hepatic CD4^+^ T cells express significant amounts of LAG3; LAG3^+^CD4^+^ T cells secrete less IFN-γ [2]. Furthermore, our data has shown LAG3 to be unchanged on other immune cells (e.g. NK, NKT, B cells, dendritic cells, eosinophil, neutrophil, monocyte) during chronic *E*. *multilocularis* infection. Early studies suggested that LAG3 could be present on many different immune cells, in which having more LAG3 is associated with certain aspects of worse disease [34–36]. This study showed that LAG3 deficiency leads to a relative skewing of naive CD4 T cells toward a Th1 phenotype, characterized by IFN-γ secretion to control metacestode growth, in mice with chronic infection and in adoptively transferred recipient mice; thus, LAG3 participates in AE disease remission, predominantly mediated by Th1 subset. Some studies have shown that LAG3-deficient T cells display increased proliferation and IFN-γ production in acute and chronic infections, and LAG3 blockade induces similar effects, further underlining the role of LAG3 as a negative regulator of the immune system [37,38].

Interestingly, we also observed that LAG3 deficiency significantly inhibited IL-4 production in hepatic and splenic CD4^+^ T cells at a late stage, suggesting that LAG3 may play a critical role in regulating Th2 differentiation in chronic *E*. *multilocularis* infection [39]. A number of studies have demonstrated that Th2 immune responses are associated with a progressive form of AE in humans and that Th1 immune responses are associated with resistance [40–42]. A previous report showed that upon stimulation, newly differentiated Th2 cells, as well as memory Th2 cells, expressed high levels of the inhibitory receptor TIGIT, and TIGIT blockade inhibited Th2 polarization and dampened Th2 responses *in vitro* and *in vivo* [43]. In addition, LAG3 expression was found at similar frequencies on all subsets of T helper cells, including Th1, Th17, Th1/Th17, and Th0/2 cells, and on memory T cells in patients with ulcerative colitis [44]. Our results also showed that LAG3 deficiency resulted in an increased percentage of CD4^+^ Tem cells. Moreover, naive CD4^+^ T cells developed rapidly into CD4^+^ Tem cells after adoptive transfer into mice and *E*. *multilocularis* challenge. Compared with CD4^+^ Tem cells that had previously formed or not been exposed to pathogens, these subsequently differentiated CD4^+^ Tem cells are considered to have a highly specific immune response against pathogens [45,46]. These results suggest that LAG3 is expressed on the Th2 subset involved in inflammation that contributes to the progression of infection, while LAG3 deficiency drives naive CD4^+^ T cells to express T-bet and develop into Th1 cells and the development of effector memory T cells following chronic *E*. *multilocularis* infection.

Furthermore, Tregs with increased expression of inhibitory receptors displayed strong immunosuppressive functions and promoted IL-10 and TGF-β1 secretion. Some studies have demonstrated that LAG3 itself is required to maintain Tregs and that LAG3 deficiency causes Tregs to exhibit reduced regulatory activity [17,18,47]. In this work, we found that LAG3 expression was predominantly found in the adaptive Treg (iTreg) lineage of AE patients, suggesting that LAG3 is primarily expressed on iTregs differentiated from peripheral naive CD4^+^ T cells rather than on natural Tregs (nTregs) generated by high-avidity interaction-based selection in the thymus [48]. Despite observations that LAG3 deficiency delays lesion growth by inhibiting Foxp3^+^ Treg and Th2 cell differentiation and promoting Th1 cell formation in the late stage of *E*. *multilocularis* infection, our data revealed a different outcome in the middle stage of infection. LAG3 deficiency increased IFN-γ and decreased IL-4 secretion by T cells, but we did not observe significant alterations in Foxp3^+^ Treg differentiation and function, which led to failure to inhibit the growth of metacestodes in the middle stage of infection. Therefore, Foxp3^+^ Treg differentiation may also be mediated by molecules other than LAG3, particularly during the middle stage of infection.

As a major cytokine secreted by Tregs, IL-10 plays a key role in limiting Th1-type responses and mediating immune tolerance [49,50]. Interestingly, our data showed that IL-10 expression by CD4^+^ T cells was significantly upregulated with LAG3 deficiency. Strikingly, we also found that adoptive transfer of naive CD4^+^ T cells from LAG3-KO donor mice to recipient mice significantly increased the IL-10 production of donor cells. In line with our findings, a previous study showed that LAG3-KO CD4^+^ T cells secrete significantly more IFN-γ and IL-10 than WT CD4^+^ T cells in murine graft-versus-host disease [51]. Additionally, LAG3 expression is increased in LAG3-KO mice compared with WT controls after prion infection but does not modify disease progression [52]. Furthermore, a study on schistosomiasis revealed that PD-1 deficiency increased IFN-γ secretion in T cells but also promoted Treg cell function, which did not result in a significant reduction in pathogen burden after schistosomiasis infection in PD-1-deficient mice [53]. These results suggest that IL-10 may play an immune-suppressive role independent of Foxp3^+^ Tregs in the middle stage of *E*. *multilocularis* infection in LAG3-KO mice. Moreover, LAG3 may enhance Treg function when the inflammatory and/or worm antigenic burden is high at a late stage of infection but not in less demanding environments and/or with the liver metacestode loads that exist in the early and middle stages of *E*. *multilocularis* infection. Earlier studies have reported similar results [34]. Further analysis is expected to reveal the actual role of LAG3 and IL-10 in the regulation of T-cell function in patients with AE.

In conclusion, our study confirms that the increased expression of LAG3 in AE patients and *E*. *multilocularis*-infected mice is associated with disease progression and CD4^+^ T-cell immune imbalance. LAG3 deficiency can restore the Th1 immune response of CD4^+^ T cells and effectively defend against parasitic infections at a late stage. Identification of the mechanism that regulates CD4^+^ T-cell responses during AE provides important information regarding protection against *E*. *multilocularis* infection and may provide a novel immunotherapeutic strategy to prevent it.

## Materials and methods

### Ethics statement

The study is approved by the ethics committee of First Affiliated Hospital of Xinjiang Medical University (S20130418-3) and all patients signed informed consent. All the procedures and experiments were approved by the Ethics Committee of the First Affiliated Hospital of Xinjiang Medical University (No: 20170809–01).

### Human subjects

A total of 27 AE patients undergoing liver resection were enrolled in this study. All AE diagnosis was confirmed by computed tomography and liver biopsy in the First Affiliated Hospital of Xinjiang Medical University, Urumqi, China. Patients with immunesuppression-associated conditions, as described in Chauchet et al., were excluded [54]. One specimen was collected close to the parasitic lesion, including the metacestode (close liver tissue [CLT], about 0.5 cm from lesion), and/or one was taken in the macroscopically normal liver distant from the lesion (distant liver tissue [DLT], at least 2 cm distant from lesion), as reported by our previous studies [6]. The number of liver tissue samples, types of measurements, and purpose of comparisons are included in Table 1. Information about AE patients in this study is listed in S1 Table.

**Table 1:** Liver samples from AE patients used for immunological studies.

			Patients with AE			
Experiment			CLT, Number of Samples	DLT, Number of Samples	Paired CLT and DLT, Number of Sample Pairs	Comparison Between Groups
Liver	IHC	LAG3	19	19	19	CLT vs. DLT
	IF	CD4	8	8	8	CLT vs. DLT
		CD8	8	8	8	CLT vs. DLT
		LAG3	8	8	8	CLT vs. DLT
	FC	LAG3	8	8	8	CLT vs. DLT

Abbreviations: IHC, immunohisto chemistry; IF, immunofluorescence; FC, flow cytometry; CLT, close liver tissue; DLT, distant liver tissue; vs, versus.

### Mice, parasite, and infection

Age-matched female C57BL/6 wild-type (WT) were purchased from Beijing Vital River Experimental Animal Technology Co. Ltd. C57BL/6 LAG3-knockout (KO) mice were obtained from Shanghai Model Organisms Center, Inc. C57BL/6 CD4 knockout (KO) mice and CD45.1 mice were kindly provided by Dr. ZheXiong Lian (Guangdong Academy of Medical Sciences, Guangzhou, China). The mice were raised in the pathogen-free environment of the animal experimental center of Xinjiang Medical University.

*E*. *multilocularis* protoscoleces (PSCs) was prepared in advance and isolated from the abdominal cavity of infected gerbils in a sterile environment. Mice were inoculated through the hepatic portal vein with live PSCs in saline as described, whereas control mice were injected with isotonic saline [2].

### Flow cytometry

Single-cell suspension was incubated in PBS buffer (PBS containing 0.2% BSA and 0.1% sodium azide) in the presence of neutralizing monoclonal antibodies against CD16:CD32 (Fc Block) for 30 min at 4°C prior to staining. Then, the cells were stained on the surface, intracellular cytokines and nucleus according to the methods in previous study [6,24]. Flow cytometer were performed, and data were analyzed. Information about the antibodies used in this study was listed in S2 Table.

### *E*. *multilocularis* antigen preparation and *in vitro* stimulation

The *E*. *multilocularis* PSCs antigen (EmP) was obtained by the previously defined method [6]. Liver lymphocytes isolated from wild type and LAG3-KO mice, were stimulated with 5 μg/ml EmP in 96 well plates at 37°C for 5 days, respectively. After stimulation, cells were removed from the plates and stained for cell surface (NK1.1, CD3, CD4, CD25), intracecellular cytokines (IFN-γ, IL-4 and IL-10) and nucleus (Foxp3), and then analyzed using Flow cytometry.

### Purification of naive CD4^+^ T cells from spleen of mice

Naive CD4^+^ T cells from spleen of LAG3-KO (CD45.2) mice, were purified by negative magnetic selection using the naive CD4^+^ T cell Isolation Kit, and subsequently separated by LS Column and separator according to the manufacturer’s protocol.

### Adoptive transfer

Splenocytes were isolated from wild type (CD45.1) and LAG3-KO (CD45.2) mice and mixed at equal ratio, and then 2×10^7^ total cells were co-transferred i.v. into *E*. *multilocularis* infected CD4-KO recipient mice. Recipients were sacrificed and CD4^+^ T cells from liver and spleen were analyzed one month post-transfer.

Naive CD4^+^T cells from spleen of LAG3-KO (CD45.2) mice were isolated and 1×10^6^ cells were transferred i.v. into wild type (CD45.1) recipient mice 24 hrs before *E*. *multilocularis* infection. Recipients were sacrificed and CD4^+^ T cells from liver and spleen were analyzed one month post-transfer.

### Statistical analysis

Statistical analysis was performed using the SPSS Statistics 25 and the results were expressed as mean ± standard deviation. Independent sample t-test was used to compare the two groups. Independent paired Student’s t tests or one-way ANOVA test was used to compare the intra group data. For all experimental results, *P* < 0.05 was considered to be significant. (p-values were expressed as follows: *p values < 0.05; **p values < 0.01; ***p values < 0.001).

## Supporting information

S1 Data Excel spreadsheet containing, in separate sheets, the raw data for Figure panels 1, 2, 3, 4, 5, 6, S2, S3, S4, S5, S6, and S7. (XLSX)

S1 File Includes: S1 to S7 Figs. S1 and S2 Tables. (DOCX)

S1 Fig Single-cell RNA-sequence defines LAG3 expression predominantly in iTreg clusters from AE patients. **(A)** UMAP clustering plot of CD4^+^ T cells derived from CLT, DLT and PB of AE patients (n = 4). **(B)** Violin plots showing the expression of LAG3 among CD4^+^ T cells clusters in CLT, DLT and PB of AE patients (n = 4). iTreg, induced-Treg cells. UMAP, uniform manifold approximation and projection. CLT, “close” liver tissue; DLT, “distant” liver tissue; PB, peripheral blood.
(TIF)

S2 Fig LAG3 is mainly expressed on Th2 and Treg cells in spleen of *E*. *multilocularis*-infected mice after week 24 infection. **(A)** Representative flow cytometry plot and percentage of IL-4, IL-10 and TGF-β1 production by CD4^+^ T cells in the spleen from mice after 24 weeks of infection (5–6 mice per group). **(B)** MFI of GATA3 expression by LAG3^+^ and LAG3^-^ CD4^+^T cells in the spleen from mice after 24 weeks of infection (5–6 mice per group). **(C)** Representative flow cytometry plot and percentage of Treg cells (CD4^+^CD25^+^Foxp3^+^) by LAG3^+^ and LAG3^-^ CD4^+^T cells in the spleen from mice after 24 weeks of infection (6 mice per group). **(D)** MFI of Ki67 expression by LAG3^+^ and LAG3^-^ CD4^+^T cells in the spleen from mice after 24 weeks of infection (5 mice per group). All data are presented as mean ± SD. **P < 0.01, ***P < 0.001, n.s., *P* > 0.05.
(TIF)

S3 Fig LAG3 deficiency delays disease progression by promoting Th1 cell differention in spleen of *E*. *multilocularis*-infected mice after week 24 infection. **(A)** Percentage and absolute numbers of CD4^+^T cells in the spleen from *E*. *multilocularis*-infected WT and LAG3-KO mice (5 mice per group). **(B)** Percentage of Tn and Tem in CD4^+^ T cells in the spleen from *E*. *multilocularis*-infected WT and LAG3-KO mice (5 mice per group). **(C, D)** Representative flow cytometry plot and percentage of IFN-γ, TNF-α, IL-4 and IL-10 production by CD4^+^ T cells in the spleen from *E*. *multilocularis*-infected WT and LAG3-KO mice (5–6 mice per group). **(E, F)** Representative flow cytometry plot and percentage of Treg cells (CD4^+^CD25^+^Foxp3^+^) in the spleen from *E*. *multilocularis*-infected WT and LAG3-KO mice (5–6 mice per group). KO, knockout; WT, wild type; Tn, naive T cells (CD44^-^CD62L^+^); Tem, effector T cells (CD44^+^CD62L^-^). All data are presented as mean ± SD. *P < 0.05, **P < 0.01, n.s., *P* > 0.05.
(TIF)

S4 Fig LAG3 deficiency limits Treg cell proliferation in a mouse model of *E*. *multilocularis* infection after week 24 infection. **(A, B)** MFI of Ki67 expresssion by LAG3^+^ and LAG3^-^ Treg cells (CD4^+^CD25^+^Foxp3^+^) in the liver and spleen from mice after 24 weeks of infection, respectively (5 mice per group). **(C, D)** MFI of Ki67 expression by Treg cells (CD4^+^CD25^+^Foxp3^+^) in the liver and spleen from WT and LAG3-KO mice after 24 weeks of infection, respectively (5–6 mice per group). KO, knockout; WT, wild type. All data are presented as mean ± SD. *P < 0.05, n.s., *P* > 0.05.
(TIF)

S5 Fig LAG3 deficiency does not delay disease progression in liver of *E*. *multilocularis*-infected mice after week 12 infection. **(A)** Representative images of metacestode tissue in liver from WT and LAG3-KO mice after 12 weeks of infection. Metacestode tissues are circled by the yellow line. **(B)** Lesion weight in liver from WT and LAG3-KO mice after 12 weeks of infection (10 mice per group). **(C)** Percentage and absolute numbers of CD4^+^T cells in the liver from WT and LAG3-KO mice after 12 weeks of infection (5–6 mice per group). **(D)** Percentage of Tn and Tem in CD4^+^ T cells in the liver from WT and LAG3-KO mice after 12 weeks of infection (5–6 mice per group). **(E, F)** Representative flow cytometry plot and percentage of IFN-γ, TNF-α, IL-4 and IL-10 production by CD4^+^ T cells in the liver from WT and LAG3-KO mice after 12 weeks of infection (5–6 mice per group). **(G, H)** Representative flow cytometry plot and percentage of Treg cells (CD4^+^CD25^+^Foxp3^+^) in the liver from WT and LAG3-KO mice after 12 weeks of infection (5–6 mice per group). KO, knockout; WT, wild type; Tn, naive T cells (CD44^-^CD62L^+^); Tem, effector T cells (CD44^+^CD62L^-^). All data are presented as mean ± SD. **P < 0.01, n.s., *P* > 0.05.
(TIF)

S6 Fig LAG3 deficiency does not delay disease progression in spleen of *E*. *multilocularis*-infected mice after week 12 infection. **(A)** Percentage and absolute numbers of CD4^+^T cells in the spleen from WT and LAG3-KO mice after 12 weeks of infection (5–6 mice per group). **(B)** Percentage of Tn and Tem in CD4^+^ T cells in the spleen from WT and LAG3-KO mice after 12 weeks of infection (5–6 mice per group). **(C, D)** Representative flow cytometry plot and percentage of IFN-γ, TNF-α, IL-4 and IL-10 production by CD4^+^ T cells in the spleen from WT and LAG3-KO mice after 12 weeks of infection (5–6 mice per group). **(E, F)** Representative flow cytometry plot and percentage of Treg (CD4^+^CD25^+^Foxp3^+^) cells in the spleen from WT and LAG3-KO mice after 12 weeks of infection (5–6 mice per group). KO, knockout; WT, wild type. All data are presented as mean ± SD. *P < 0.05, **P < 0.01, n.s., *P* > 0.05.
(TIF)

S7 Fig LAG3 deficiency enhances Th1 cells and decrease Treg upon adoptive transfer into wild-type mice followed by *E*. *multilocularis* infection. **(A)** Percentage of CD4^+^ Tn and Tem in autocells in the spleen from *E*. *multilocularis*-infected WT recipient mice (CD45.1) with transferred by LAG3-KO (CD45.2) cells. **(B, C)** Representative flow cytometry plot and percentage of IFN-γ, IL-4 and IL-10 production by CD4^+^ T cells in the spleen from *E*. *multilocularis*-infected WT recipient mice (CD45.1) with transferred by LAG3-KO (CD45.2) cells. **(D, E)** Representative flow cytometry plot and percentage of Treg cells(CD4^+^CD25^+^Foxp3^+^) in the spleen from *E*. *multilocularis*-infected WT recipient mice (CD45.1) with transferred by LAG3-KO (CD45.2) cells. KO, knockout; WT, wild type; Tn, naive T cells. All data are presented as mean ± SD. **P < 0.01, ***P < 0.001, n.s., *P* > 0.05.
(TIF)

S1 Table Baseline clinical characteristics of AE patients studied. (DOCX)

S2 Table Antibodies for flow cytometry. (DOCX)
