# Supplementary material for: Upregulation of LAG3 modulates the immune imbalance of CD4+ T-cell subsets and exacerbates disease progression in patients with alveolar echinococcosis and a mouse model
Source: PLoS Pathog. 2023 May 12;19(5):e1011396. doi: 10.1371/journal.ppat.1011396 (PMC10208502; doi:10.1371/journal.ppat.1011396)
Supplement: S1 File — (DOCX) [file ppat.1011396.s002.docx]

**Supporting information for**

**Upregulation of LAG3 modulates the immune imbalance of CD4^+^ T-cell subsets and exacerbates disease progression in patients with alveolar echinococcosis and a mouse model**

**Supporting file 2 includes:**

**S1 to S7 Figs**

**S1 and S2 Tables**

**S1 Fig. Single-cell RNA-sequence defines LAG3 expression predominantly in iTreg clusters from AE patients**

**(A)** UMAP clustering plot of CD4^+^ T cells derived from CLT, DLT and PB of AE patients (n=4). **(B)** Violin plots showing the expression of LAG3 among CD4^+^ T cells clusters in CLT, DLT and PB of AE patients (n=4). iTreg, induced-Treg cells. UMAP, uniform manifold approximation and projection. CLT, “close” liver tissue; DLT, “distant” liver tissue; PB, peripheral blood.

**S2 Fig. LAG3 is mainly expressed on Th2 and Treg cells in spleen of *E. multilocularis*-infected mice after week 24 infection**

**(A)** Representative flow cytometry plot and percentage of IL-4, IL-10 and TGF-β1 production by CD4^+^ T cells in the spleen from mice after 24 weeks of infection (5-6 mice per group). **(B)** MFI of GATA3 expression by LAG3^+^ and LAG3^-^ CD4^+^T cells in the spleen from mice after 24 weeks of infection (5-6 mice per group). **(C)** Representative flow cytometry plot and percentage of Treg cells (CD4^+^CD25^+^Foxp3^+^) by LAG3^+^ and LAG3^-^ CD4^+^T cells in the spleen from mice after 24 weeks of infection (6 mice per group). **(D)** MFI of Ki67 expression by LAG3^+^ and LAG3^-^ CD4^+^T cells in the spleen from mice after 24 weeks of infection (5 mice per group). All data are presented as mean ± SD. ***P* < 0.01, ****P* < 0.001, n.s., *P* > 0.05.

**S3 Fig. LAG3 deficiency delays disease progression by promoting Th1 cell differention in spleen of *E. multilocularis*-infected mice after week 24 infection**

**(A)** Percentage and absolute numbers of CD4^+^T cells in the spleen from *E. multilocularis*-infected WT and LAG3-KO mice (5 mice per group). **(B)** Percentage of Tn and Tem in CD4^+^ T cells in the spleen from *E. multilocularis*-infected WT and LAG3-KO mice (5 mice per group). **(C, D)** Representative flow cytometry plot and percentage of IFN-γ, TNF-α, IL-4 and IL-10 production by CD4^+^ T cells in the spleen from *E. multilocularis*-infected WT and LAG3-KO mice (5-6 mice per group). **(E, F)** Representative flow cytometry plot and percentage of Treg cells (CD4^+^CD25^+^Foxp3^+^) in the spleen from *E. multilocularis*-infected WT and LAG3-KO mice (6 mice per group). KO, knockout; WT, wild type; Tn, naive T cells (CD44^-^CD62L^+^); Tem, effector T cells (CD44^+^CD62L^-^). All data are presented as mean ± SD. **P* < 0.05, ***P* < 0.01, n.s., *P* > 0.05.

**S4 Fig. LAG3 deficiency limits Treg cell proliferation in a mouse model of *E. multilocularis* infection after week 24 infection**

**(A, B)** MFI of Ki67 expresssion by LAG3^+^ and LAG3^-^ Treg cells (CD4^+^CD25^+^Foxp3^+^) in the liver and spleen from mice after 24 weeks of infection, respectively (5 mice per group). **(C, D)** MFI of Ki67 expression by Treg cells (CD4^+^CD25^+^Foxp3^+^) in the liver and spleen from WT and LAG3-KO mice after 24 weeks of infection, respectively (5-6 mice per group). KO, knockout; WT, wild type. All data are presented as mean ± SD. **P* < 0.05, n.s., *P* > 0.05.

**S5 Fig. LAG3 deficiency does not delay disease progression in liver of *E. multilocularis*-infected mice after week 12 infection**

**(A)** Representative images of metacestode tissue in liver from WT and LAG3-KO mice after 12 weeks of infection. Metacestode tissues are circled by the yellow line. **(B)** Lesion weight in liver from WT and LAG3-KO mice after 12 weeks of infection (10 mice per group). **(C)** Percentage and absolute numbers of CD4^+^T cells in the liver from WT and LAG3-KO mice after 12 weeks of infection (5-6 mice per group). **(D)** Percentage of Tn and Tem in CD4^+^ T cells in the liver from WT and LAG3-KO mice after 12 weeks of infection (5-6 mice per group). **(E, F)** Representative flow cytometry plot and percentage of IFN-γ, TNF-α, IL-4 and IL-10 production by CD4^+^ T cells in the liver from WT and LAG3-KO mice after 12 weeks of infection (5-6 mice per group). **(G, H)** Representative flow cytometry plot and percentage of Treg cells (CD4^+^CD25^+^Foxp3^+^) in the liver from WT and LAG3-KO mice after 12 weeks of infection (5-6 mice per group). KO, knockout; WT, wild type; Tn, naive T cells (CD44^-^CD62L^+^); Tem, effector T cells (CD44^+^CD62L^-^). All data are presented as mean ± SD. ***P* < 0.01, n.s., *P* > 0.05.

**S6 Fig. LAG3 deficiency does not delay disease progression in spleen of *E. multilocularis*-infected mice after week 12 infection**

**(A)** Percentage and absolute numbers of CD4^+^T cells in the spleen from WT and LAG3-KO mice after 12 weeks of infection (5-6 mice per group). **(B)** Percentage of Tn and Tem in CD4^+^ T cells in the spleen from WT and LAG3-KO mice after 12 weeks of infection (5-6 mice per group). **(C, D)** Representative flow cytometry plot and percentage of IFN-γ, TNF-α, IL-4 and IL-10 production by CD4^+^ T cells in the spleen from WT and LAG3-KO mice after 12 weeks of infection (5-6 mice per group). **(E,F)** Representative flow cytometry plot and percentage of Treg (CD4^+^CD25^+^Foxp3^+^) cells in the spleen from WT and LAG3-KO mice after 12 weeks of infection (5-6 mice per group). KO, knockout; WT, wild type. All data are presented as mean ± SD. **P* < 0.05, ***P* < 0.01, n.s., *P* > 0.05.

**S7 Fig. LAG3 deficiency enhances Th1 cells and decrease Treg upon adoptive transfer into wild-type mice followed by *E. multilocularis* infection**

**(A)** Percentage of CD4^+^ Tn and Tem in autocells in the spleen from *E. multilocularis*-infected WT recipient mice (CD45.1) with transferred by LAG3-KO (CD45.2) cells. **(B, C)** Representative flow cytometry plot and percentage of IFN-γ, IL-4 and IL-10 production by CD4^+^ T cells in the spleen from *E. multilocularis*-infected WT recipient mice (CD45.1) with transferred by LAG3-KO (CD45.2) cells. **(D, E)** Representative flow cytometry plot and percentage of Treg cells(CD4^+^CD25^+^Foxp3^+^) in the spleen from *E. multilocularis*-infected WT recipient mice (CD45.1) with transferred by LAG3-KO (CD45.2) cells. KO, knockout; WT, wild type; Tn, naive T cells. All data are presented as mean ± SD. ***P* < 0.01, ****P* < 0.001, n.s., *P* > 0.05.

**S1 Table. Baseline clinical characteristics of AE patients studied**

**S2 Table. Antibodies for flow cytometry**
