# Supplementary figures and images for: Upregulation of LAG3 modulates the immune imbalance of CD4+ T-cell subsets and exacerbates disease progression in patients with alveolar echinococcosis and a mouse model
Source: PLoS Pathog. 2023 May 12;19(5):e1011396. doi: 10.1371/journal.ppat.1011396 (PMC10208502; doi:10.1371/journal.ppat.1011396)

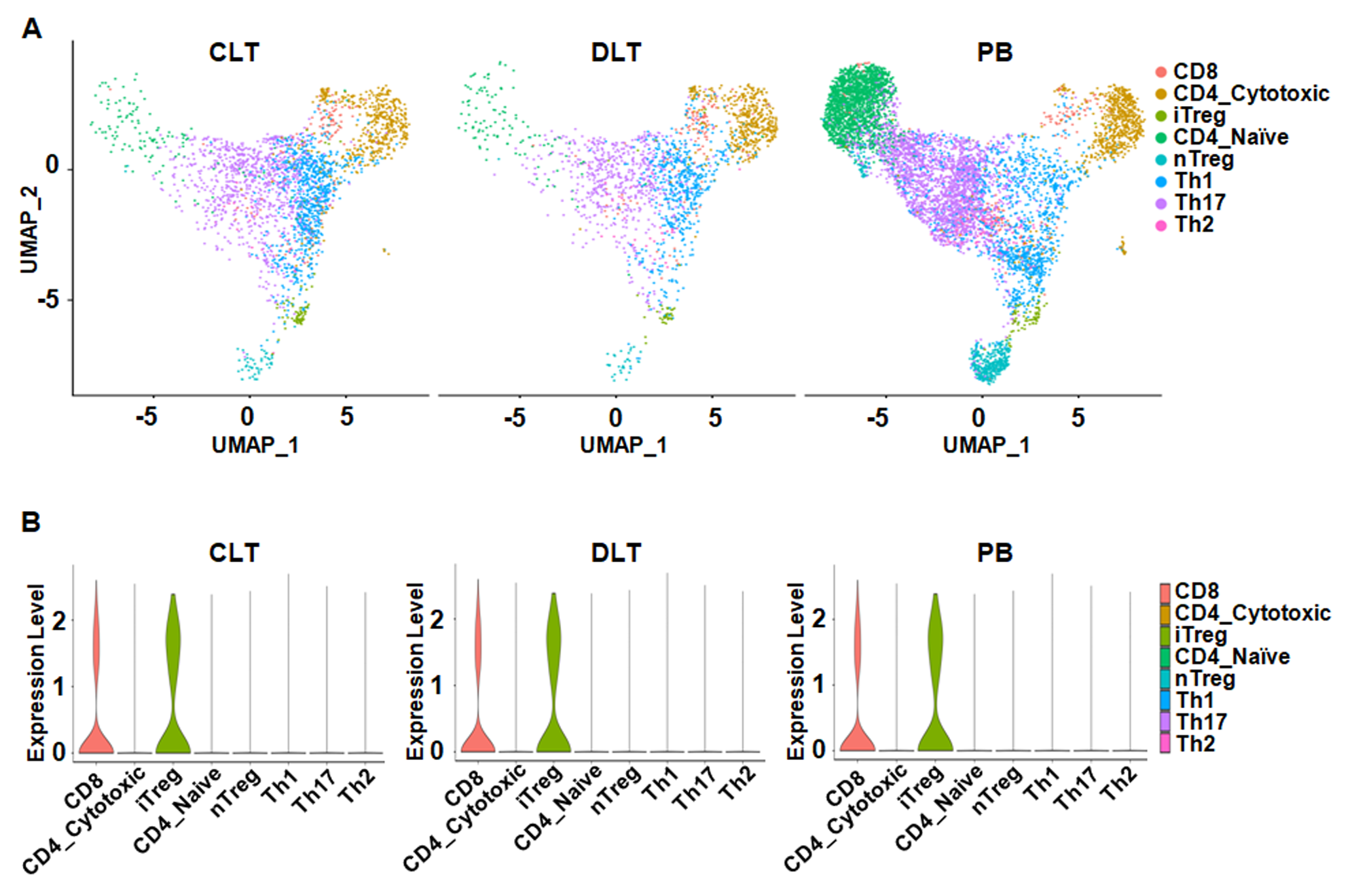

Supplement: S1 Fig — (A) UMAP clustering plot of CD4+ T cells derived from CLT, DLT and PB of AE patients (n = 4). (B) Violin plots showing the expression of LAG3 among CD4+ T cells clusters in CLT, DLT and PB of AE patients (n = 4). iTreg, induced-Treg cells. UMAP, uniform manifold approximation and projection. CLT, “close” liver tissue; DLT, “distant” liver tissue; PB, peripheral blood. (TIF) [file ppat.1011396.s003.tif]

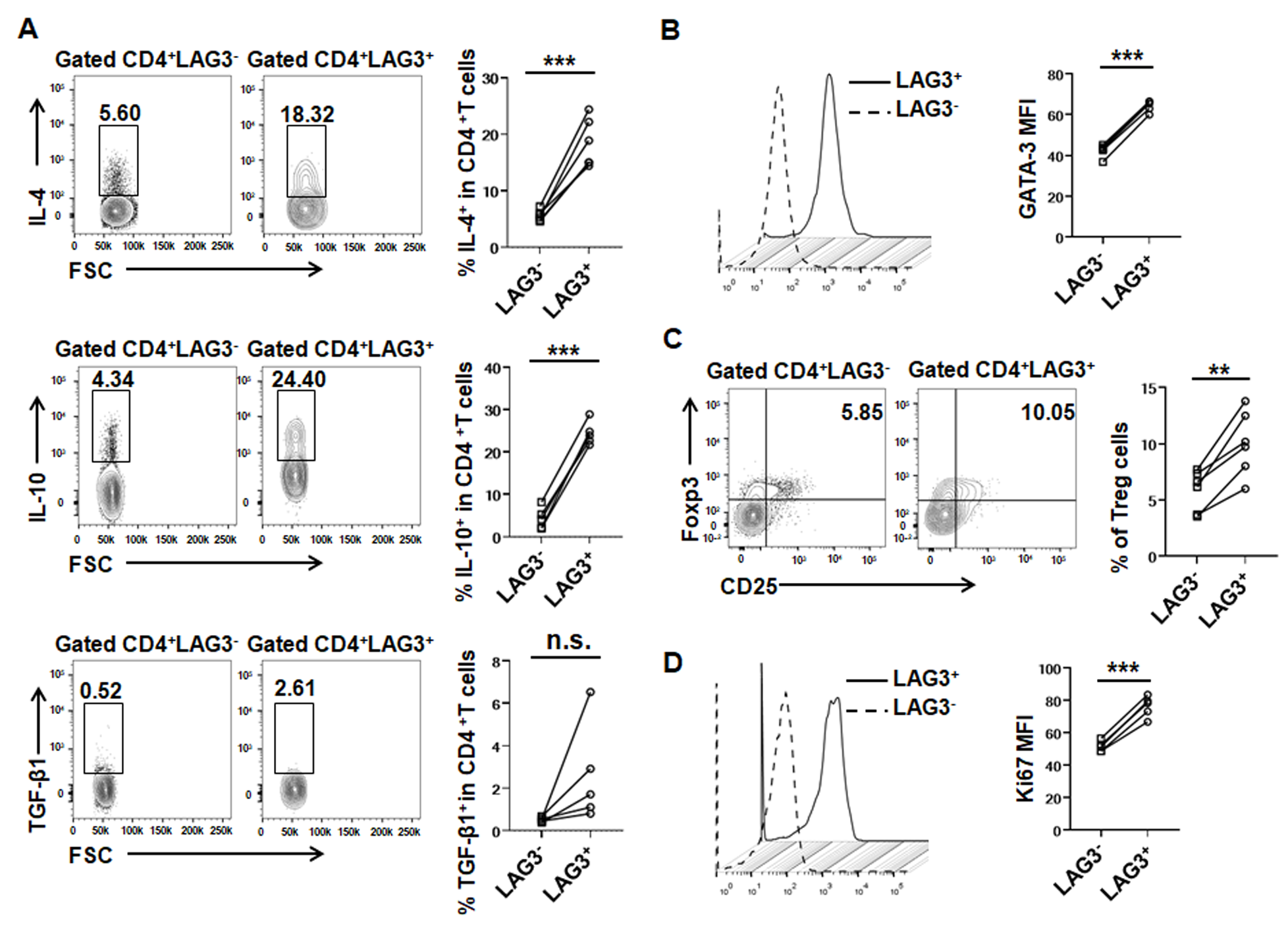

Supplement: S2 Fig — (A) Representative flow cytometry plot and percentage of IL-4, IL-10 and TGF-β1 production by CD4+ T cells in the spleen from mice after 24 weeks of infection (5–6 mice per group). (B) MFI of GATA3 expression by LAG3+ and LAG3- CD4+T cells in the spleen from mice after 24 weeks of infection (5–6 mice per group). (C) Representative flow cytometry plot and percentage of Treg cells (CD4+CD25+Foxp3+) by LAG3+ and LAG3- CD4+T cells in the spleen from mice after 24 weeks of infection (6 mice per group). (D) MFI of Ki67 expression by LAG3+ and LAG3- CD4+T cells in the spleen from mice after 24 weeks of infection (5 mice per group). All data are presented as mean ± SD. **P < 0.01, ***P < 0.001, n.s., P > 0.05. (TIF) [file ppat.1011396.s004.tif]

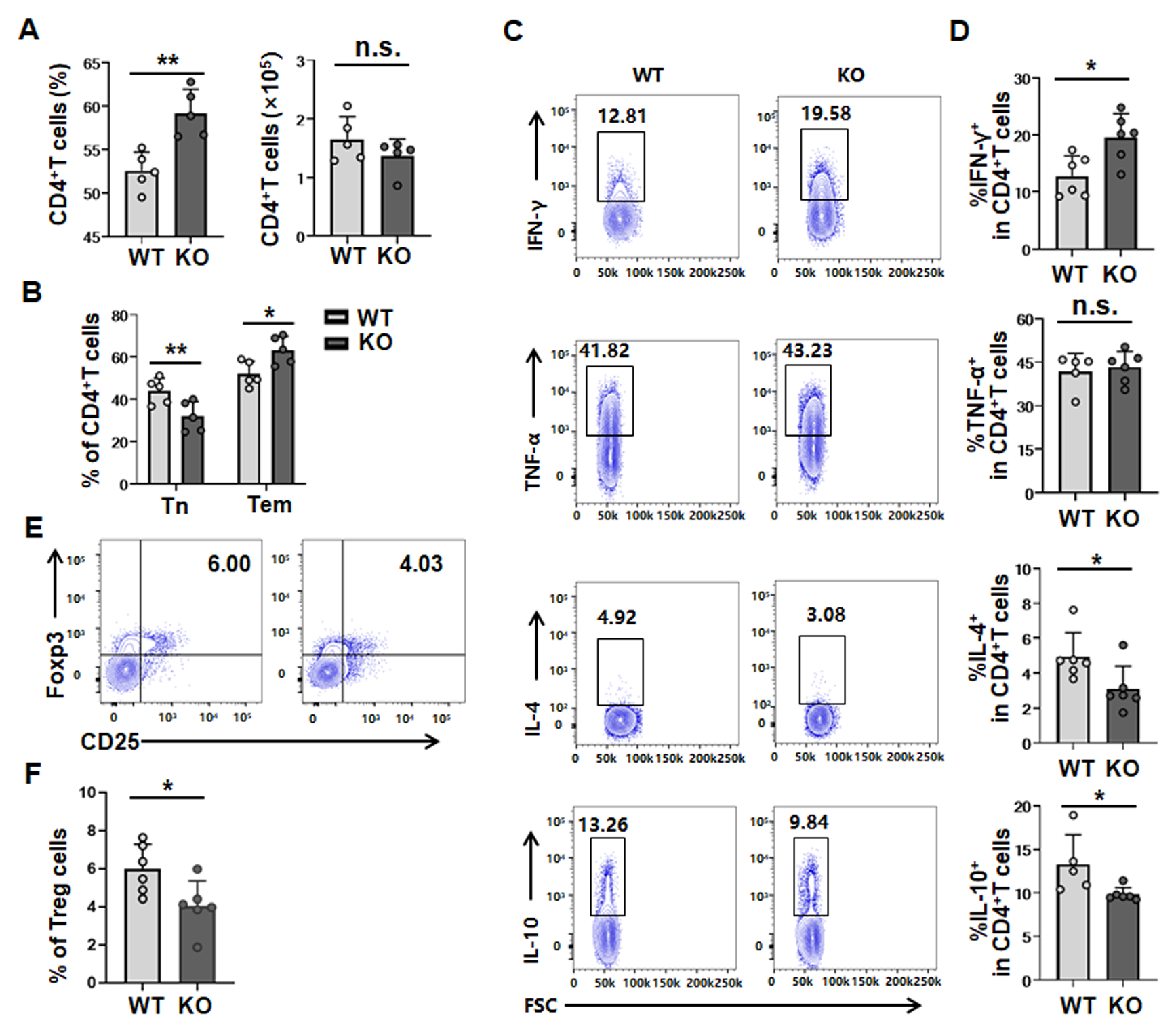

Supplement: S3 Fig — (A) Percentage and absolute numbers of CD4+T cells in the spleen from E. multilocularis-infected WT and LAG3-KO mice (5 mice per group). (B) Percentage of Tn and Tem in CD4+ T cells in the spleen from E. multilocularis-infected WT and LAG3-KO mice (5 mice per group). (C, D) Representative flow cytometry plot and percentage of IFN-γ, TNF-α, IL-4 and IL-10 production by CD4+ T cells in the spleen from E. multilocularis-infected WT and LAG3-KO mice (5–6 mice per group). (E, F) Representative flow cytometry plot and percentage of Treg cells (CD4+CD25+Foxp3+) in the spleen from E. multilocularis-infected WT and LAG3-KO mice (5–6 mice per group). KO, knockout; WT, wild type; Tn, naive T cells (CD44-CD62L+); Tem, effector T cells (CD44+CD62L-). All data are presented as mean ± SD. *P < 0.05, **P < 0.01, n.s., P > 0.05. (TIF) [file ppat.1011396.s005.tif]

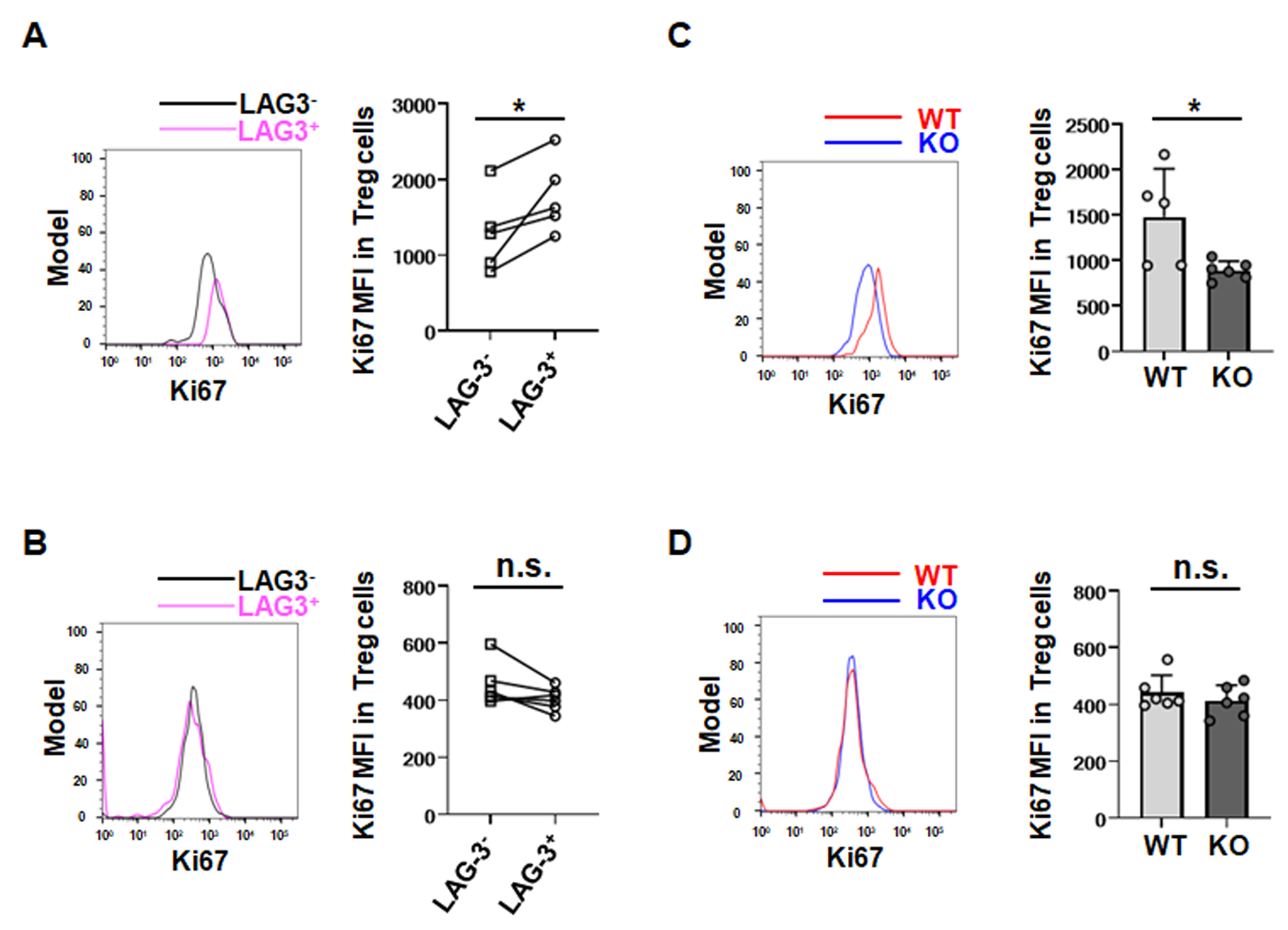

Supplement: S4 Fig — (A, B) MFI of Ki67 expresssion by LAG3+ and LAG3- Treg cells (CD4+CD25+Foxp3+) in the liver and spleen from mice after 24 weeks of infection, respectively (5 mice per group). (C, D) MFI of Ki67 expression by Treg cells (CD4+CD25+Foxp3+) in the liver and spleen from WT and LAG3-KO mice after 24 weeks of infection, respectively (5–6 mice per group). KO, knockout; WT, wild type. All data are presented as mean ± SD. *P < 0.05, n.s., P > 0.05. (TIF) [file ppat.1011396.s006.tif]

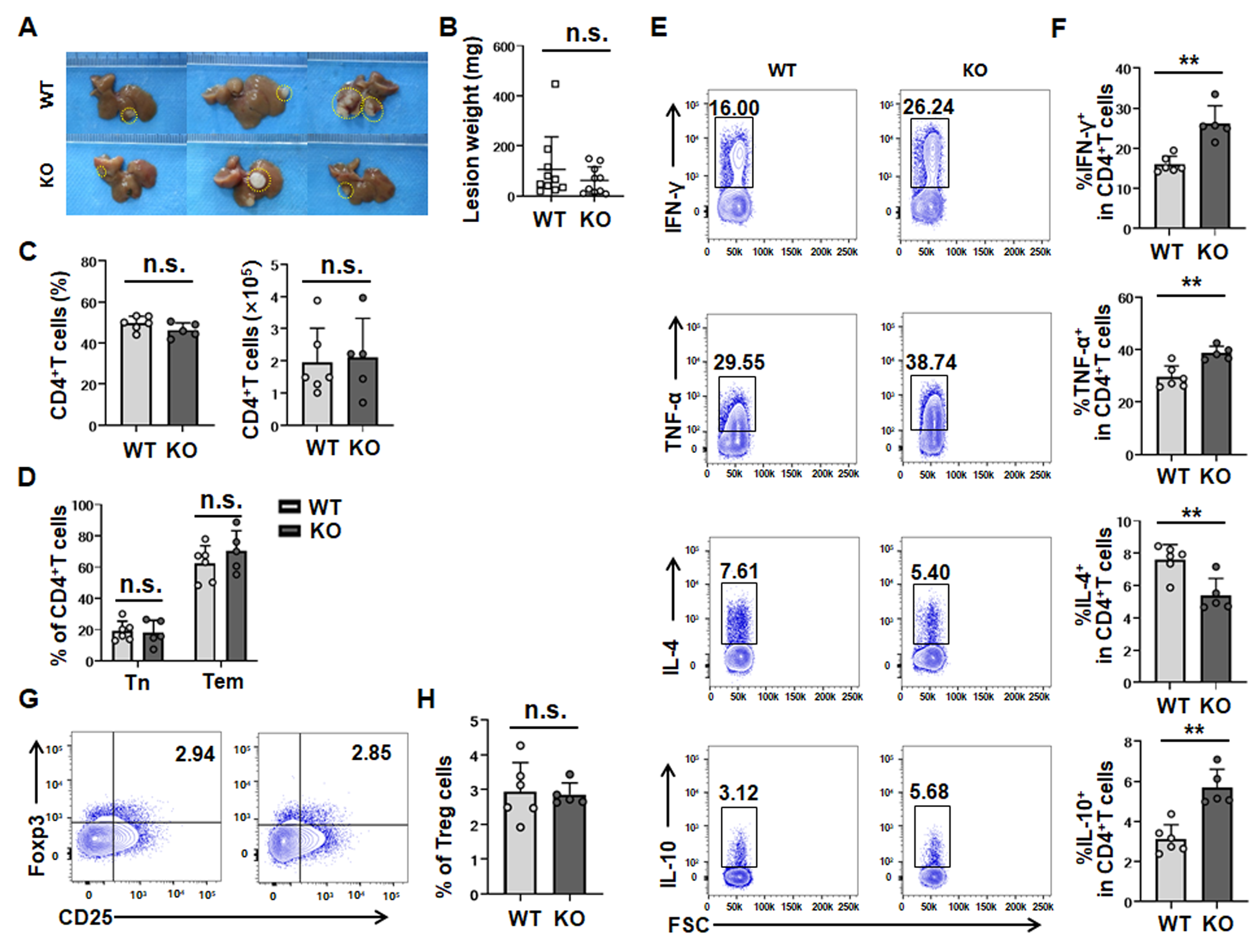

Supplement: S5 Fig — (A) Representative images of metacestode tissue in liver from WT and LAG3-KO mice after 12 weeks of infection. Metacestode tissues are circled by the yellow line. (B) Lesion weight in liver from WT and LAG3-KO mice after 12 weeks of infection (10 mice per group). (C) Percentage and absolute numbers of CD4+T cells in the liver from WT and LAG3-KO mice after 12 weeks of infection (5–6 mice per group). (D) Percentage of Tn and Tem in CD4+ T cells in the liver from WT and LAG3-KO mice after 12 weeks of infection (5–6 mice per group). (E, F) Representative flow cytometry plot and percentage of IFN-γ, TNF-α, IL-4 and IL-10 production by CD4+ T cells in the liver from WT and LAG3-KO mice after 12 weeks of infection (5–6 mice per group). (G, H) Representative flow cytometry plot and percentage of Treg cells (CD4+CD25+Foxp3+) in the liver from WT and LAG3-KO mice after 12 weeks of infection (5–6 mice per group). KO, knockout; WT, wild type; Tn, naive T cells (CD44-CD62L+); Tem, effector T cells (CD44+CD62L-). All data are presented as mean ± SD. **P < 0.01, n.s., P > 0.05. (TIF) [file ppat.1011396.s007.tif]

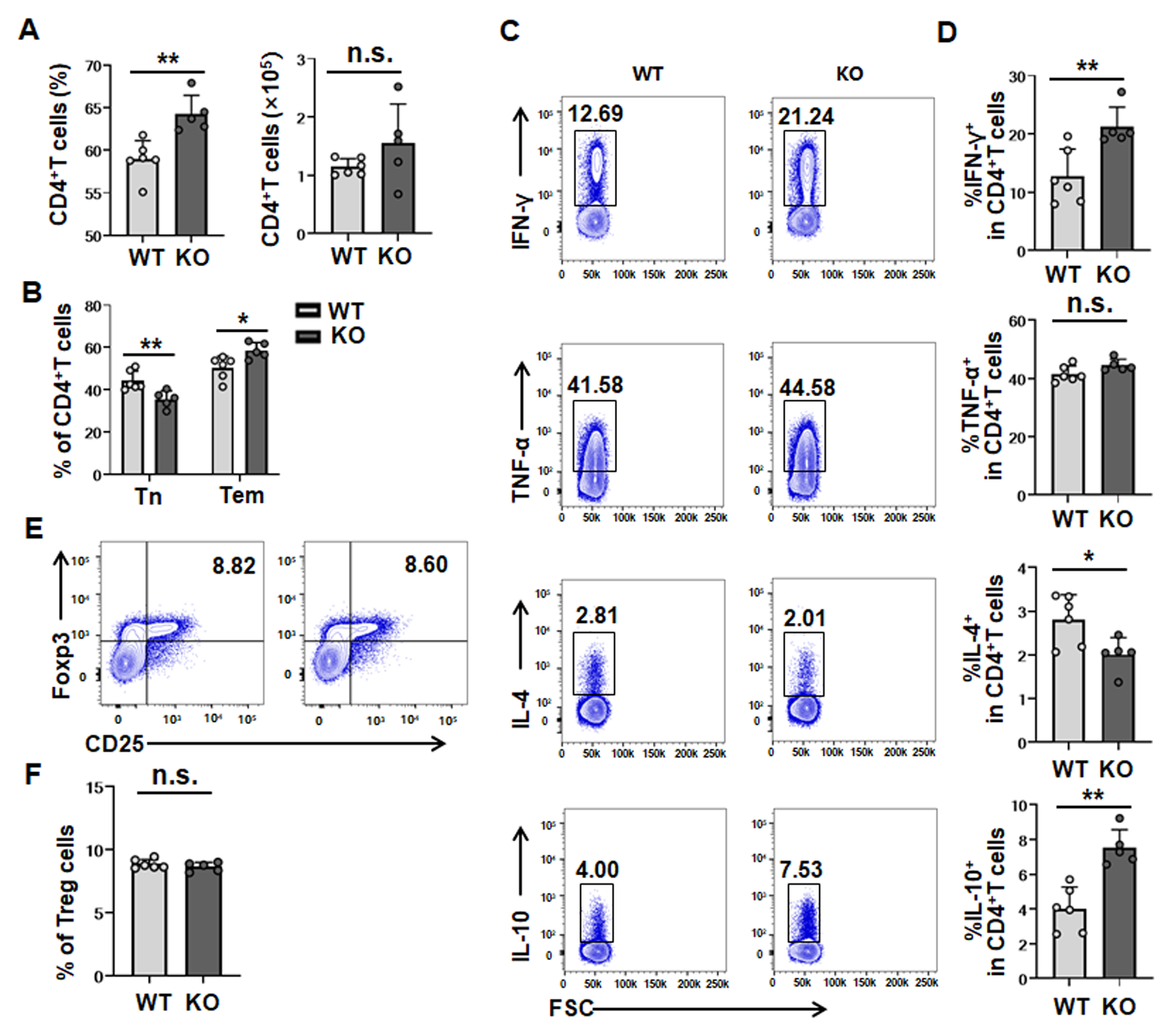

Supplement: S6 Fig — (A) Percentage and absolute numbers of CD4+T cells in the spleen from WT and LAG3-KO mice after 12 weeks of infection (5–6 mice per group). (B) Percentage of Tn and Tem in CD4+ T cells in the spleen from WT and LAG3-KO mice after 12 weeks of infection (5–6 mice per group). (C, D) Representative flow cytometry plot and percentage of IFN-γ, TNF-α, IL-4 and IL-10 production by CD4+ T cells in the spleen from WT and LAG3-KO mice after 12 weeks of infection (5–6 mice per group). (E, F) Representative flow cytometry plot and percentage of Treg (CD4+CD25+Foxp3+) cells in the spleen from WT and LAG3-KO mice after 12 weeks of infection (5–6 mice per group). KO, knockout; WT, wild type. All data are presented as mean ± SD. *P < 0.05, **P < 0.01, n.s., P > 0.05. (TIF) [file ppat.1011396.s008.tif]

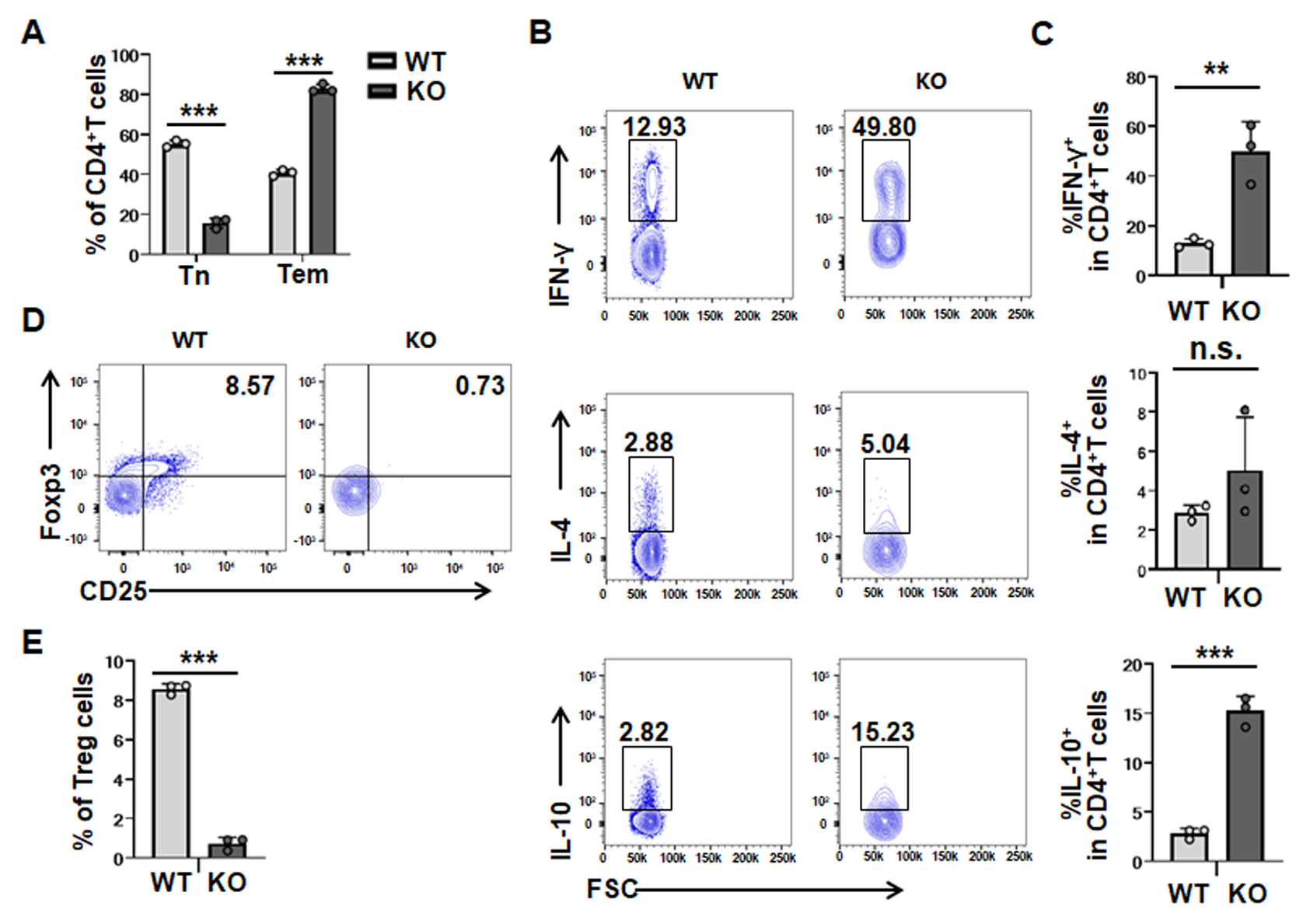

Supplement: S7 Fig — (A) Percentage of CD4+ Tn and Tem in autocells in the spleen from E. multilocularis-infected WT recipient mice (CD45.1) with transferred by LAG3-KO (CD45.2) cells. (B, C) Representative flow cytometry plot and percentage of IFN-γ, IL-4 and IL-10 production by CD4+ T cells in the spleen from E. multilocularis-infected WT recipient mice (CD45.1) with transferred by LAG3-KO (CD45.2) cells. (D, E) Representative flow cytometry plot and percentage of Treg cells(CD4+CD25+Foxp3+) in the spleen from E. multilocularis-infected WT recipient mice (CD45.1) with transferred by LAG3-KO (CD45.2) cells. KO, knockout; WT, wild type; Tn, naive T cells. All data are presented as mean ± SD. **P < 0.01, ***P < 0.001, n.s., P > 0.05. (TIF) [file ppat.1011396.s009.tif]
