# Supplementary material for: Upregulation of LAG3 modulates the immune imbalance of CD4+ T-cell subsets and exacerbates disease progression in patients with alveolar echinococcosis and a mouse model
Source: PLoS Pathog. 2023 May 12;19(5):e1011396. doi: 10.1371/journal.ppat.1011396 (PMC10208502; doi:10.1371/journal.ppat.1011396)
Supplement: S1 Table — (DOCX) [file ppat.1011396.s010.docx]

**S1 Table. Baseline clinical characteristics of AE patients studied**

| **Patient No.** | **Age (years)** | **Gender (F/M)** | **PMN stage** | **ALP (U/L)** | **ALT (U/L)** | **AST (U/L)** | **Em 2+** | **Characterization of lesions (location, diameter [cm])** | **Treatment before surgery (mo.)** | **Metastasis** | **PET before surgery (TBR)** | **Surgical approach** |
| --- | --- | --- | --- | --- | --- | --- | --- | --- | --- | --- | --- | --- |
| 1 | 15 | F | P4N0M0 | 114 | 9 | 13 | + | LL, 11.0×6.8;  RL, 11.8×9.7 | N | N | 3.7 | ELRA |
| 2 | 26 | F | P3N0M1 | 83 | 16 | 23 | + | LL+RL, 9.5×8.7 | N | N | 4.6 | ELRA |
| 3 | 32 | M | P3N0M0 | 258 | 45 | 35 | N | LL, 4.6×4.3;  RL, 19.9×15.3 | N | N | 5.3 | ELRA |
| 4 | 36 | F | P2N0M0 | 130 | 16 | 23 | + | RL, 10.5×6.3 | N | N | 2.7 | RHR |
| 5 | 41 | M | P2N0M0 | 214 | 60 | 47 | － | LL+RL, 20.7×15.7 | 4, ABZ | N | 3.9 | ELRA |
| 6 | 32 | F | P2N1M0 | 461 | 36 | 39 | + | LL+RL, 16.8×13.4 | 6, ABZ | pancreas | 3.3 | RHR |
| 7 | 38 | F | P4N1M0 | 100 | 8 | 19 | + | RL, 10.5×10.7 | 48, TM | kidney | 6.1 | ELRA |
| 8 | 26 | F | P4N0M0 | 407 | 125 | 101 | － | LL, 15.3×10.1 | 1, ABZ | N | 2.8 | ELRA |
| 9 | 44 | M | P3N0M0 | 205 | 25 | 22 | + | LL+RL, 5.2×5.7 | 5, ABZ | N | 4.6 | RHR |
| 10 | 34 | M | P4N1M1 | 456 | 27 | 36 | － | RL+CL, 10.2×8.6 | N | N | 2.9 | ELRA |
| 11 | 47 | F | P4N0M0 | 140 | 17 | 18 | N | LL, 5.9×5.4; 13.3×8.7;  RL, 3.0×2.1 | 2nd | N | 4.4 | ELRA |
| 12 | 29 | M | P3N0M1 | 122 | 11 | 18 | + | LL+RL, 15.2×12.1 | N | lung | 4.4 | RHR |
| 13 | 36 | F | P4N0M0 | 195 | 12 | 22 | + | RL, 12.3×10.4 | N | N | 4.5 | ELRA |
| 14 | 18 | M | P2N1M0 | 246 | 12 | 16 | + | LL, 4.4×4.4;  RL, 11.0×11.4 | N | N | 2.5 | ELRA |
| 15 | 30 | F | P4N1M0 | 232 | 20 | 24 | + | RL, 20.4×16.1 | 84, ABZ | N | 3.9 | ELRA |
| 16 | 37 | M | P3N1M1 | 137 | 48 | 21 | + | LL+RL, 3.5×2.7 | 108, ABZ, 2nd | lung, kidney | 3.1 | ELRA |
| 17 | 32 | F | P3N0M1 | 78 | 20 | 23 | + | RL, 7.1×9.4;  LL+RL, 17.4×10.7 | 72, ABZ | lung | 9.4 | ELRA |
| 18 | 31 | M | P3N1M0 | 78 | 22 | 27 | N | LL+RL, 14.0×13.6 | 2nd | N | 5.6 | RHR |
| 19 | 48 | F | P4N1M0 | 274 | 49 | 33 | N | LL, 12.6×10.9 | N | N | 2.8 | RHR |
| 20 | 37 | M | P3N0M0 | 127 | 10 | 17 | + | CL, 6.7×7.9 | 2nd | N | 1.9 | ELRA |
| 21 | 27 | F | P3N0M0 | 276 | 16 | 23 | + | RL, 11.5×18.3 | N | N | 4.1 | RHR |
| 22 | 29 | M | P1N1M1 | 87 | 10 | 14 | + | LL+RL, 15.0×14.1 | N | lung, kidney | 11.9 | PHR |
| 23 | 19 | F | P2N1M0 | 172 | 10 | 22 | + | LL+RL, 10.6×8.6 | N | N | N | RHR |
| 24 | 33 | M | P4N1M0 | 454 | 90 | 101 | + | LL+RL, 15.0×10.9 | N | N | N | RHR |
| 25 | 62 | M | P3N0M0 | 199 | 199 | 32 | + | RL, 8.4×5.1 | 2nd | N | 2.6 | RHR |
| 26 | 76 | M | P1N1M0 | 306 | 40 | 24 | + | CL+RL, 12.4×11.6 | N | lung | N | N |
| 27 | 45 | M | P3N0M0 | 67 | 42 | 19 | + | RL, 3.6×3.6 | N | N | 5.4 | ELRA |
| Abbreviations: F, female; M, male; LL, left liver lobe; RL, right liver lobe; CL, caudate liver lobe; ALP, alkaline phosphatase; ALT, alanine aminotransferase; AST, aspartate aminotransferase; +, positive; ―, negative; ABZ: Albendazole; TM: Tibetan medicine; 2nd: Second surgery; RHR, Radical hepatic resection; ELRA, Ex vivo liver resection and autotransplantation; PHR, Palliative hepatic resection; N, none; NA, not available. | | | | | | | | | | | | |
