# Supplementary material for: Upregulation of LAG3 modulates the immune imbalance of CD4+ T-cell subsets and exacerbates disease progression in patients with alveolar echinococcosis and a mouse model
Source: PLoS Pathog. 2023 May 12;19(5):e1011396. doi: 10.1371/journal.ppat.1011396 (PMC10208502; doi:10.1371/journal.ppat.1011396)
Supplement: S2 Table — (DOCX) [file ppat.1011396.s011.docx]

**S2 Table. Antibodies for flow cytometry**

| **Reagent or Resource** | **Clone** | **Source** | **Catalog Number** |
| --- | --- | --- | --- |
| Purified anti-mouse CD16/32 |  | BioLegend | Cat# 101302 |
| Anti-mouse CD3-FITC | 17A2 | BioLegend | Cat# 100204 |
| Anti-mouse CD45.1-FITC | A20 | BioLegend | Cat# 110713 |
| Anti-mouse CD69-PE | H1.2F3 | BioLegend | Cat# 104508 |
| Anti-mouse GATA3-PE | 16E10A23 | BioLegend | Cat# 653804 |
| Anti-mouse IL-4-PE | 11B11 | BioLegend | Cat# 504104 |
| Anti-mouse LAG-3-PE | C9B7W | BioLegend | Cat# 125207 |
| Anti-mouse IL-10-PE/Dazzle 594 | JES5-16E3 | BioLegend | Cat# 505034 |
| Anti-mouse T-bet-PE/Dazzle 594 | 4B10 | BioLegend | Cat# 644828 |
| Anti-mouse CD4-PerCP/Cy5.5 | GK1.5 | BioLegend | Cat# 100433 |
| Anti-mouse CD45.2-PerCP/Cy5.5 | 104 | BioLegend | Cat# 109827 |
| Anti-mouse CD62L-PerCP/Cy5.5 | MEL-14 | BioLegend | Cat# 104431 |
| Anti-mouse LAG-3-PerCP/Cy5.5 | C9B7W | BioLegend | Cat# 125212 |
| Anti-mouse Ki67-PerCP/Cy5.5 | 16A8 | BioLegend | Cat# 652424 |
| Anti-mouse TNF-α-PerCP/Cy5.5 | MP6-XT22 | BioLegend | Cat# 506322 |
| Anti-mouse CD3-APC | 17A2 | BioLegend | Cat# 100236 |
| Anti-mouse CD44-APC | IM7 | BioLegend | Cat# 103012 |
| Anti-mouse IFN-γ-APC | XMG1.2 | BioLegend | Cat# 505810 |
| Anti-mouse TGF-β1-APC | TW7-16B4 | BioLegend | Cat# 141406 |
| Anti-mouse CD3-APC-Cy7 | 17A2 | BioLegend | Cat# 100222 |
| Anti-mouse CD4-APC-Cy7 | GK1.5 | BioLegend | Cat# 100413 |
| Anti-mouse CD25-APC-Cy7 | PC61 | BioLegend | Cat# 102026 |
| Anti-mouse CD44-APC-Cy7 | IM7 | BioLegend | Cat# 103028 |
| Anti-mouse CD4-PB | GK1.5 | BioLegend | Cat# 100428 |
| Anti-mouse CD25-Brilliant Violet 421 | PC61 | BioLegend | Cat# 102033 |
| Anti-mouse LAG3-Brilliant Violet 421 | C9B7W | BioLegend | Cat# 125221 |
| Anti-mouse CD3-Brilliant Violet 510 | 17A2 | BioLegend | Cat# 100233 |
| Anti-mouse CD62L-Brilliant Violet 510 | MEL-14 | BioLegend | Cat# 104441 |
| Anti-mouse Foxp3-Alexa Fluor 647 | MF-14 | BioLegend | Cat# 126408 |
| Anti-mouse CD4-Brilliant Violet 605 | GK1.5 | BioLegend | Cat# 100451 |
